# Oxidative degradation of dihydrofolate reductase increases CD38-mediated ferroptosis susceptibility

**DOI:** 10.1038/s41419-022-05383-7

**Published:** 2022-11-09

**Authors:** Yingying Ma, Meiqi Yi, Weixuan Wang, Xiaohui Liu, Qingtao Wang, Chongdong Liu, Yuling Chen, Haiteng Deng

**Affiliations:** 1 MOE Key Laboratory of Bioinformatics, Center for Synthetic and Systematic Biology, School of Life Sciences, Tsinghua University, 100084 Beijing, China; 2 BeiGene (Beijing) Co., Ltd., 100084 Beijing, China; 3 Institute of Chinese Medicine, Guangdong Pharmaceutical University, 510006 Guangzhou, China; 4 Beijing Chao-yang Hospital, Capital Medical University, 100043 Beijing, China

**Keywords:** Cell death, Post-translational modifications

## Abstract

High expression of CD38 in tissues is a characteristic of aging, resulting in a decline in nicotinamide adenine dinucleotide (NAD) and increasing cellular reactive oxygen species (ROS). However, whether CD38 increases susceptibility to ferroptosis remains largely unexplored. Our previous study showed that CD38 overexpression decreased dihydrofolate reductase (DHFR). In the present study, we confirmed that high expression of CD38 increased ROS levels and induced DHFR degradation, which was prevented by nicotinamide mononucleotide (NMN) replenishment. We further revealed that ROS-mediated sulfonation on Cys7 of DHFR induced its degradation via the autophagy and non-canonical proteasome pathways. Mutation of Cys7 to alanine abolished ROS-induced DHFR degradation. Moreover, oxidative degradation of DHFR was responsible for the increased ferroptosis susceptibility of cells in which CD38 was highly expressed. We also found that CD38 expression was higher in bone-marrow-derived macrophages (BMDMs) from aged mice than those from young mice, while the DHFR level was lower. Consequently, we demonstrated that BMDMs from aged mice were more susceptible to ferroptosis that can be reverted by NMN replenishment, suggesting that CD38 high expression rendered cells more susceptible to ferroptosis. Taken together, these results indicated that CD38-mediated NAD^+^ decline promoted DHFR oxidative degradation, thus resulting in increased cellular susceptibility to ferroptosis and suggesting that NMN replenishment may protect macrophages from ferroptosis in aged mice.

## Introduction

Nicotinamide adenine dinucleotide (NAD), found in all living cells, is involved in redox reactions and various biological processes such as circadian rhythm and inflammation. As an essential co-factor of redox enzymes and a substrate for deacetylases, NAD^+^ declines during chronological aging resulting in mitochondrial dysfunction, oxidative stress, DNA damage, and cognitive impairment [1, 2]. As the main NAD-degrading enzyme in mammalian tissues and cells, CD38 mainly catalyzes NAD^+^ to adenosine diphosphate ribose (ADPR) and only a small part of NAD^+^ was converted to cyclic adenosine diphosphate ribose (cADPR) by CD38 [3]. The expression level and activity of CD38 have been shown to increase with age in various tissues and macrophages, thereby resulting in age-related NAD^+^ decline. Consequently, CD38 inhibition or knockout has been proven to preserve NAD^+^ levels in tissues during aging and to increase lifespan, healthspan, and glucose tolerance [4–6]. Replenishment of NAD^+^ precursors including nicotinamide mononucleotide (NMN), nicotinamide riboside (NR), nicotinamide (NAM), niacin (NA), or inhibition of NAD-consuming enzymes has also been shown to prolong healthspan and to treat age-related disorders (ARDs) [7, 8].

Ferroptosis is a new form of regulated cell death different from apoptosis, pyroptosis, and necrosis and is characterized by iron-dependent lipid peroxidation [9, 10]. The direct cause of ferroptosis is the accumulated lipid peroxides produced by the reaction of polyunsaturated fatty acids (PUFAs) on the membrane and hydroxyl radical, which is the product of the Fenton reaction catalyzed by ferrous ion [11, 12]. The mechanisms of ferroptosis are mainly grouped into five aspects, i.e., (i) inhibition of cysteine/glutamate antiporter (System X_C_^−^), (ii) depletion of glutathione or inhibition of phospholipid hydroperoxide glutathione peroxidase 4 (GPX4), (iii) excessive iron accumulation, especially ferrous ion, (iv) the decrease of lipid peroxide reducers including tetrahydrobiopterin (BH_4_), coenzyme Q10 (CoQ_10_), and (v) excessive accumulation of lipid peroxides [13–15]. In addition, ferroptosis has been reported to play a pivotal role in aging and diverse diseases such as aging-related neurodegenerative disorders, cancers, and ischemia-reperfusion due to iron accumulation and increased oxidative stress [16–18].

CD38 high expression aggravated cellular ROS levels, which may increase the susceptibility to ferroptosis. However, few studies have been reported on the relationship between CD38 expression and ferroptosis. Our previous results have shown that NAD^+^ decline caused by CD38 high expression triggered ROS-mediated degradation of 15-hydroxyprostaglandin dehydrogenase (15-PGDH). We also noticed that the level of dihydrofolate reductase (DHFR) was significantly decreased in CD38-overexpression cells from the proteomic analysis [19]. As a newly discovered negative regulator of ferroptosis, DHFR catalyzes the reduction of dihydrobiopterin (BH_2_) to BH_4_, which specifically reduces lipid peroxides thus inhibiting ferroptosis or alleviating cellular susceptibility to ferroptosis [20, 21]. Therefore, we proposed that CD38 overexpression increased the susceptibility to ferroptosis by causing oxidative degradation of DHFR.

In the present study, we established a cell line in which CD38 was overexpressed and we demonstrated that high expression of CD38 increased cellular ROS levels and ferroptosis susceptibility. On the other hand, NMN supplementation prevented the oxidative degradation of DHFR that decreased ferroptosis susceptibility. We further showed that the bone marrow-derived macrophages (BMDMs) from aged mice had higher CD38 expression levels than those from young mice, rendering them more susceptible to ferroptosis. Taken together, our work established a new link between the high expression of CD38 and ferroptosis.

## Results

### CD38 high expression induces the oxidative degradation of DHFR

**Fig. 1:**
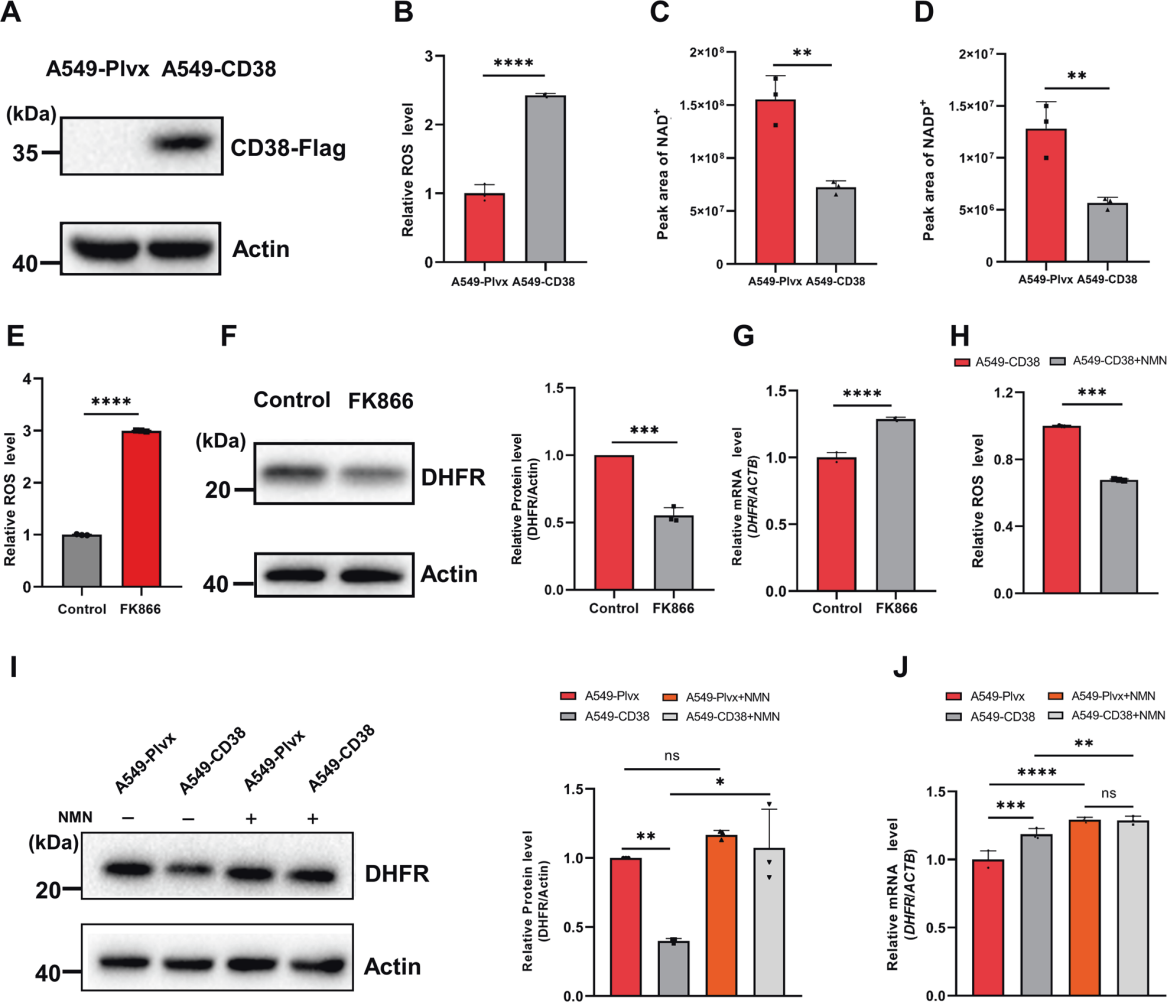
High expression of CD38 causes oxidative degradation of DHFR. **A** Western blot analysis confirmed the overexpression of CD38 in A549-CD38 cells compared with A549-Plvx cells. **B** Relative ROS levels in A549-Plvx and A549-CD38 cells (*n* = 3). **C** and **D** NAD^+^ and NADP^+^ levels in A549-Plvx and A549-CD38 cells were determined by peak areas from the metabolomics analysis (*n* = 3). **E** Relative ROS levels of A549 cells treated with or without 100 nM FK866 for 12 h (*n* = 3). **F** A549 cells were treated with or without 100 nM FK866 for 12 h. Protein levels of DHFR and Actin (loading control) were analyzed by western blot. Graphs represent the quantification of the blots (*n* = 3). **G** The relative transcription levels of DHFR in untreated and 100 nM FK866-treated A549 cells (*n* = 3). **H** The relative ROS levels in A549-CD38 cells treated with or without 1 mM NMN for 12 h (*n* = 3). **I** A549-Plvx and A549-CD38 cells were treated with or without 1 mM NMN. Protein levels of DHFR and Actin (loading control) were analyzed by western blot. Graphs represent the quantification of the blots (*n* = 3). **J** The relative transcription levels of DHFR in A549-Plvx and A549-CD38 cells treated with or without 1 mM NMN (*n* = 3). Data were shown as mean ± SD and analyzed by Student’s *t*-test or one-way ANOVA test. *p < 0.05, **p < 0.01, ***p < 0.001, ****p < 0.0001.

We established a cell model to mimic the decreased NAD^+^ levels during aging by overexpressing N-terminal truncated CD38 in A549 cells (A549-CD38 cells) as previously reported [19]. The CD38 overexpression was confirmed by western blotting (Fig. 1A). ROS levels were significantly elevated in A549-CD38 cells (Fig. 1B) while cellular NAD^+^ and NADP^+^ levels were reduced by about 55% determined by the metabolomics analysis (Fig. 1C and D). According to the proteomic results published previously [19], we noticed that the protein level of DHFR was significantly reduced in A549-CD38 cells compared to A549-Plvx cells, which was further confirmed via western blotting (Fig. 1I). However, we found that the transcription levels of DHFR were slightly increased in A549-CD38 cells (Fig. 1J), suggesting that the decrease of DHFR was due to its protein degradation process. Nicotinamide phosphoribosyltransferase (NAMPT) is the rate-limiting enzyme in the mammalian NAD^+^ synthetic salvage pathway, which catalyzes the conversion of nicotinamide (NAM) and 5-phosphoribosyl-1-pyrophosphate to NMN. To investigate whether NAD^+^ reduction was responsible for the decrease in DHFR, we treated A549 cells with FK866, an inhibitor of NAMPT. Our results demonstrated that FK866 treatment increased ROS levels in A549 cells and caused the decline of DHFR protein levels simultaneously (Fig. 1E and F). However, its mRNA level was not decreased (Fig. 1G), which demonstrated that NAD^+^ decline caused ROS-dependent DHFR degradation.

NAD^+^ precursors, NMN, NAM, NR, and NA, have been shown to boost intracellular NAD^+^ levels [22–24]. To explore whether NMN has an effect on DHFR expression levels, we treated A549-Plvx and A549-CD38 cells with NMN, respectively. We found that NMN treatment increased the protein levels of DHFR and decreased ROS levels in A549-CD38 cells, meanwhile (Fig. 1I and H). The transcription levels of DHFR were only slightly increased after NMN treatment (Fig. 1J), which suggested that NMN replenishment could prevent DHFR degradation via increasing cellular NAD^+^ levels and decreasing ROS levels at the same time. These results further confirmed the oxidative degradation of DHFR was induced by NAD^+^ decline. Our previous studies showed that NAD^+^ decline caused by CD38 overexpression lead to the oxidative degradation of 15-PGDH [19]. Therefore, these results indicated that CD38 overexpression may also cause the oxidative degradation of DHFR.

### The oxidative degradation of DHFR is dependent on the autophagy and non-canonical proteasome pathways

**Fig. 2:**
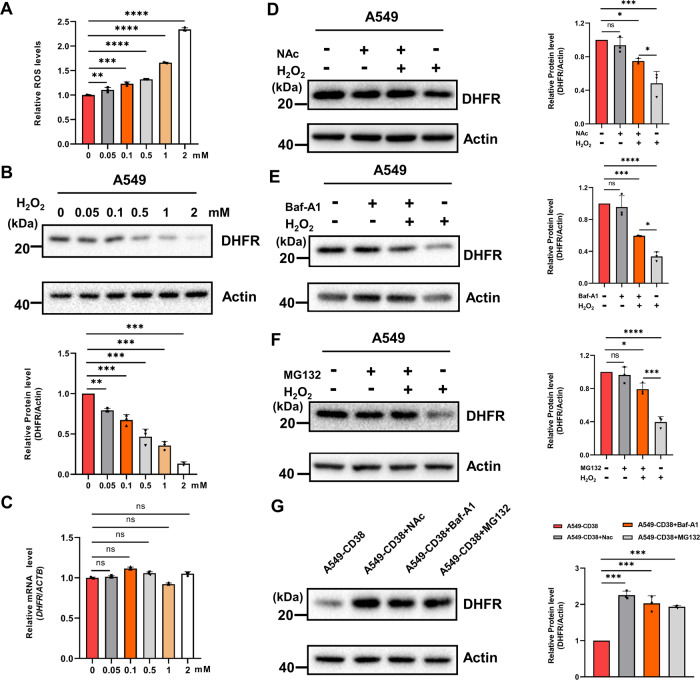
The oxidative degradation of DHFR is mediated by the autophagy and non-canonical proteasome pathways. **A** Relative ROS levels were measured in A549 cells treated with different concentrations of H_2_O_2_ (0, 0.05, 0.1, 0.5, 1, 2 mM) for 12 h (*n* = 3). **B** A549 cells were treated with different concentrations of H_2_O_2_ (0, 0.05, 0.1, 0.5, 1, 2 mM) for 12 h. Protein levels of DHFR and Actin (loading control) were analyzed by western blot. Graphs represent the quantification of the blots (*n* = 3). **C** The transcription levels of DHFR in A549 cells treated with different concentrations of H_2_O_2_ (0, 0.05, 0.1, 0.5, 1, 2 mM) for 12 h (*n* = 3). **D** Western blot images of DHFR and Actin (loading control) in untreated and 1 mM H_2_O_2_-treated A549 cells pre-treated with 6 mM NAc for 6 h or not. Graphs represent the quantification of the blots (*n* = 3). **E** Western blot images of DHFR and Actin (loading control) in untreated and 1 mM H_2_O_2_-treated A549 cells co-treated with 100 nM Baf-A1 or not. Graphs represent the quantification of the blots (*n* = 3). **F** Western blot images of DHFR and Actin (loading control) in untreated and 1 mM H_2_O_2_-treated A549 cells pre-treated with 10 μM MG132 for 4 h or not. Graphs represent the quantification of the blots (*n* = 3). **G** Western blot images of DHFR and Actin (loading control) in DMSO, NAc (6 mM, 6 h), Baf-A1 (100 nM, 6 h), and MG132 (10 μM, 4 h)-treated A549-CD38 cells. Graphs represent the quantification of the blots (*n* = 3). Data were shown as mean ± SD and analyzed by one-way ANOVA test. *p < 0.05, **p < 0.01, ***p < 0.001, ****p < 0.0001.

To further confirm the oxidative degradation of DHFR, we treated A549 cells with different concentrations of hydrogen peroxide (H_2_O_2_) for 12 h and found that the ROS levels increased progressively and the protein levels of DHFR decreased gradually with the increase of H_2_O_2_ concentrations (Fig. 2A and B), while its transcription levels were not significantly changed (Fig. 2C).

To further explore the mechanisms underlying the oxidative degradation of DHFR, we found that treatment with N-acetylcysteine (NAc) for 6 h prior to H_2_O_2_ treatment mitigated ROS-induced DHFR degradation (Fig. 2D), confirming that ROS caused DHFR degradation. Bafilomycin A1 (Baf-A1), a known inhibitor of autophagy, partially blocked the ROS-induced DHFR degradation (Fig. 2E), suggesting that the oxidative degradation of DHFR was dependent on the autophagy pathway. Furthermore, MG132, a classical proteasome inhibitor, was used to pretreat A549 cells for 4 h before H_2_O_2_ treatment in order to determine whether the oxidative degradation of DHFR was proteasome-dependent. Indeed, ROS-induced DHFR degradation was also inhibited by MG132, indicating that the oxidative degradation of DHFR was also dependent on the proteasome pathways (Fig. 2F). However, ubiquitination of DHFR was undetected, suggesting that DHFR oxidative degradation did not occur through the canonical ubiquitination-proteasome pathway (data not shown). Besides, NAc, Baf-A1, and MG132 treatment could also increase the DHFR protein levels in A549-CD38 cells (Fig. 2G), which further indicated that NAD^+^ decline caused by CD38 high expression induced the autophagy and non-canonical proteasome pathway-dependent DHFR oxidative degradation.

### ROS-caused sulfonation of cysteine7 in DHFR leads to its degradation

**Fig. 3:**
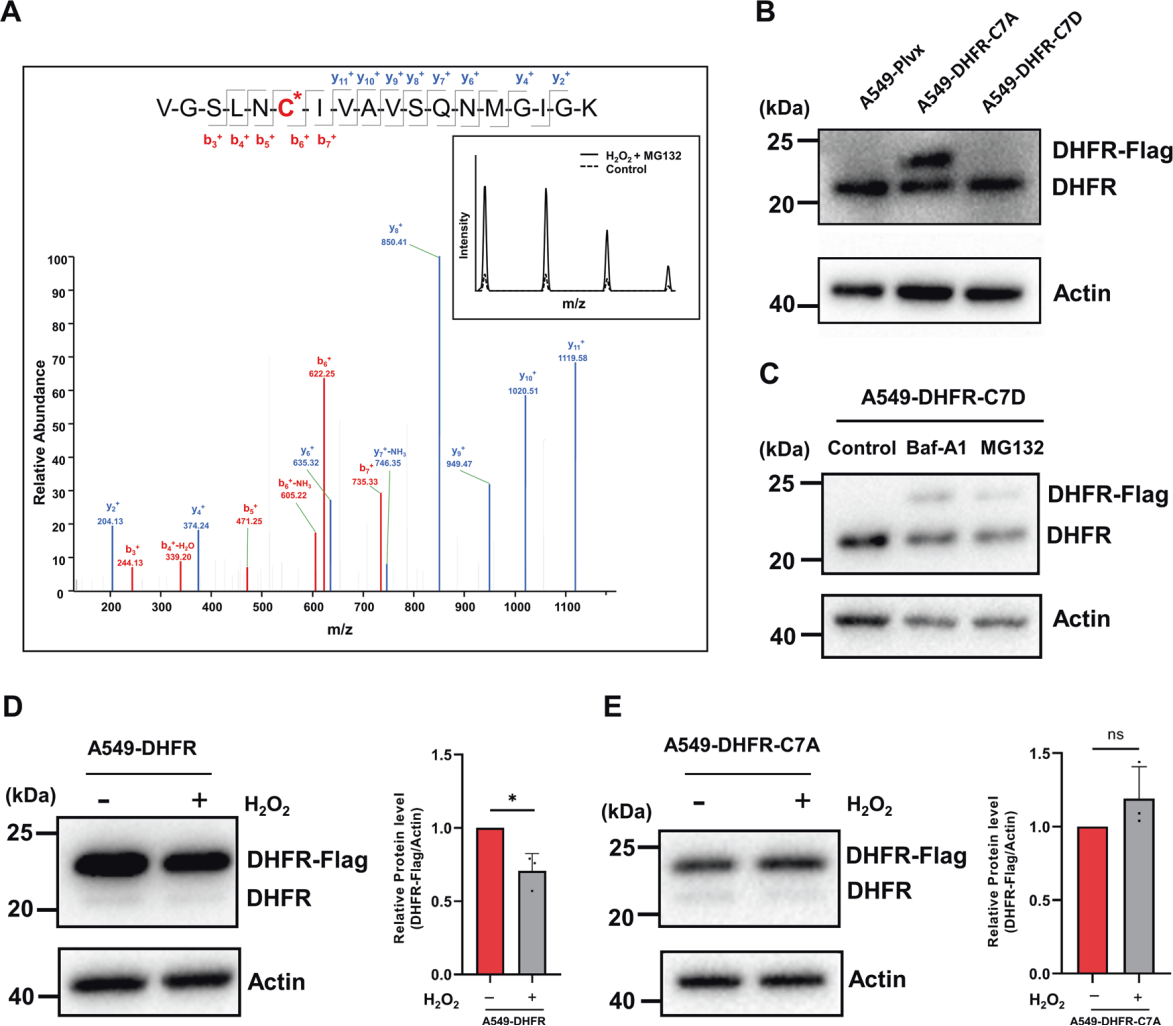
The sulfonation of Cys7 in DHFR induces its oxidative degradation. **A** The mass spectrometry spectrum of the peptide containing sulfonated Cys7 in DHFR, with the inset illustrating the ion intensity of the peptide from untreated (Control) and H_2_O_2_ treated A549-DHFR cells with pre-treatment of MG132 (H_2_O_2_ + MG132). **B** Western blot images of DHFR, Flag-tagged DHFR, and Actin in A549-Plvx, A549-DHFR-C7A, and A549-DHFR-C7D cells. **C** Western blot images of DHFR-C7D protein in DMSO, Baf-A1 (100 nM, 6 h) treated, and MG132 (10 μM, 4 h)-treated A549-C7D cells. **D** and **E** Western blot analysis of DHFR and Flag-tagged DHFR proteins from untreated and H_2_O_2_-treated A549-DHFR and A549-DHFR-C7A cells, respectively. Graphs represent the quantification of the blots (*n* = 3). Data were shown as mean ± SD and analyzed by Student’s *t*-test. *p < 0.05, **p < 0.01, ***p < 0.001, ****p < 0.0001.

Human DHFR protein consists of eight β-sheets, constituting the rigid structure of protein molecule, and four α-helices, forming the binding sites of the substrate and coenzyme, and free loop structures, connecting β-sheets and α-helices [25–27]. DHFR from different species shares high structural similarity [28]. There is only one cysteine residue in DHFR at position 7 (Cys7) and DHFR contains no disulfide bonds [29]. To figure out whether cysteine oxidation in DHFR led to its degradation, we established a stable cell line in which DHFR was overexpressed in A549 cells (A549-DHFR cells) (Fig. S1A and S1B). We found that the level of sulfonation on Cys7 in DHFR in A549-DHFR cells under H_2_O_2_ treatment was about 4.5 times higher than that in the untreated cells by immunoprecipitation followed by LC–MS/MS analysis, as confirmed by the MS/MS spectrum of the sulfonated peptide (Fig. 3A). Cys7 is located in β-sheet a of DHFR and these results indicated the sensitivity of Cys7 to oxidative stress, which might cause the conformational change of DHFR and lead to its degradation.

To further confirm whether Cys7 oxidation caused its degradation, we carried out site-directed mutagenesis of Cys7. We constructed cell lines in which Cys7 was mutated to alanine (A549-DHFR-C7A cells) or to aspartic acid (A549-DHFR-C7D cells) to eliminate Cys7 oxidation or to mimic the sulfonation of Cys7, respectively (Fig. S1C). Interestingly, only the Flag-tagged DHFR-C7A protein was detected while the Flag-tagged DHFR-C7D protein was barely detectible (Fig. 3B), which further proved that sulfonation of Cys7 in DHFR caused its oxidative degradation. Moreover, MG132 and Baf-A1 treatment indeed increased the level of Flag-tagged DHFR-C7D protein (Fig. 3C), which further confirmed that the oxidative degradation of DHFR was dependent on the autophagy and non-canonical proteasome pathways. Besides, the protein level of Flag-tagged DHFR protein was decreased under H_2_O_2_ treatment, which could be abolished in A549-DHFR-C7A cells thus demonstrating that Cys7 residue was crucial for the ROS-mediated DHFR degradation (Fig. 3D and E).

### CD38 high expression increases ferroptosis susceptibility via DHFR reduction

**Fig. 4:**
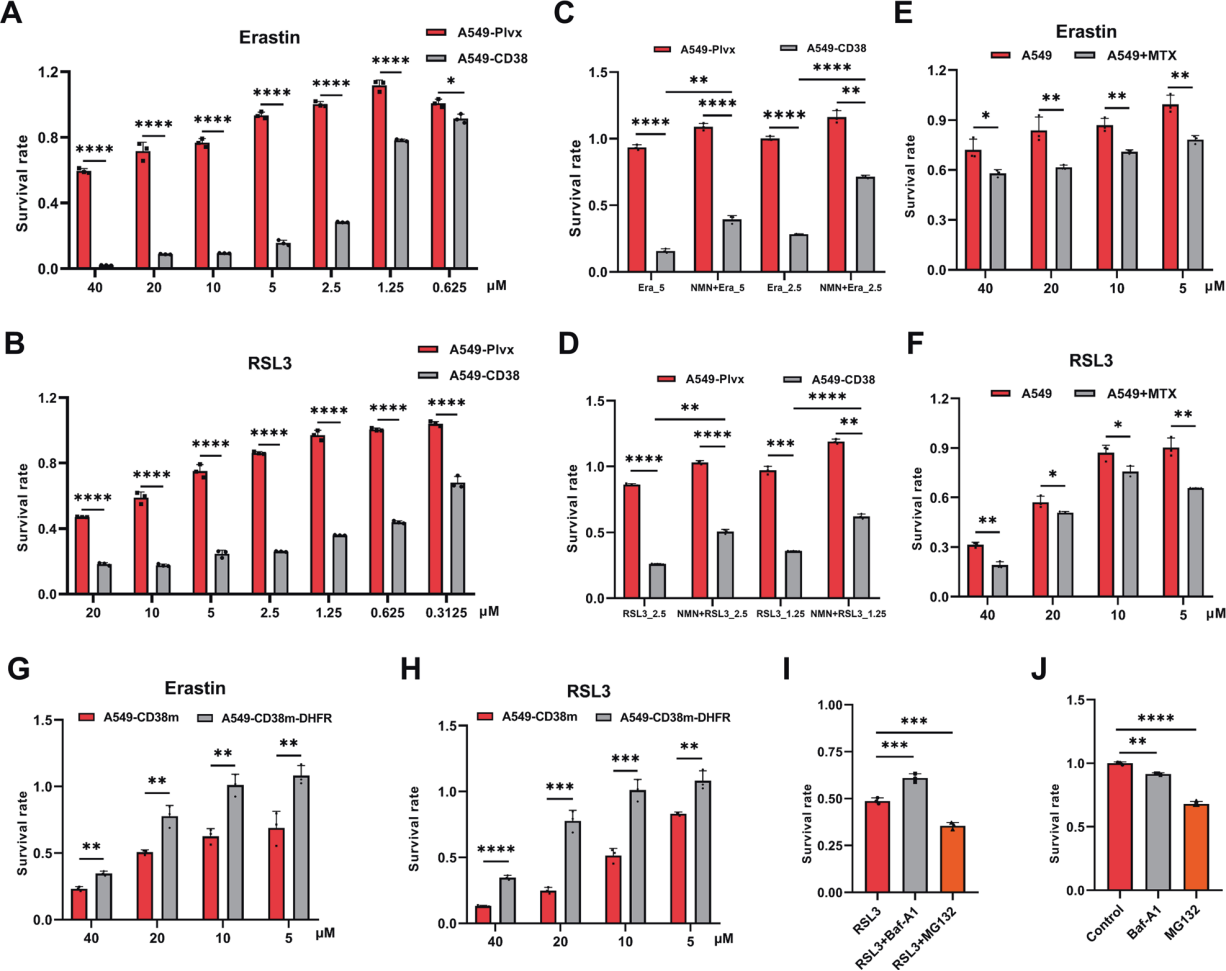
High expression of CD38 increases ferroptosis susceptibility. **A** and **B** The survival rate of A549-Plvx and A549-CD38 cells treated with different concentrations of Erastin (**A**) or RSL3 (**B**) (*n* = 3). **C** and **D** The survival rate of A549-Plvx and A549-CD38 cells treated with Erastin (5, 2.5 μM) or RSL3 (2.5, 1.25 μM) with or without 1 mM NMN co-treatment for 12 h (*n* = 3). **E** and **F** The survival rate of A549 cells under Erastin or RSL3 treatment co-treated with or without 100 nM methotrexate (MTX) (*n* = 3). **G** and **H** The survival rate of A549-CD38 cells under Erastin or RSL3 treatment before or after replenishing DHFR (*n* = 3). **I** The survival rate of A549-CD38 cells treated with RSL3 (0.625 μM, 12 h), RSL3 (0.625 μM) + Baf-A1 (100 nM, co-treatment with RSL3 for 12 h), and RSL3 (0.625 μM) + MG132 (10 μM, pre-treatment for 4 h) (*n* = 3). **J** The survival rate of A549-CD38 cells treated with DMSO (Control), Baf-A1 (100 nM), and MG132 (10 μM, pre-treatment for 4 h) for 12 h (*n* = 3). Data were shown as mean ± SD and analyzed by Student’s *t*-test or one-way ANOVA test. *p < 0.05, **p < 0.01, ***p < 0.001, ****p < 0.0001.

Considering that DHFR inhibits ferroptosis by catalyzing the reduction of BH_2_ to the lipid peroxide reducer BH_4_, we compared the susceptibility of A549-Plvx and A549-CD38 cells to ferroptosis inducers. Erastin and RSL3 are classic ferroptosis inducers that induce ferroptosis by inhibiting the System X_C_^−^ and GPX4, respectively [30, 31]. The survival of A549-CD38 cells was significantly lower than that of A549-Plvx cells when treated with Erastin or RSL3, suggesting that high expression of CD38 increased the susceptibility to ferroptosis (Fig. 4A and B).

We have found that NMN treatment prevented the oxidative degradation of DHFR in A549-CD38 cells (Fig. 1I). Therefore, we further examined the effect of NMN on ferroptosis susceptibility. Indeed, the ferroptosis induced by Erastin or RSL3 could be significantly alleviated by NMN in A549-CD38 cells according to the results of survival rate (Fig. 4C and D), suggesting that NMN suppressed the oxidative degradation of DHFR to alleviate cellular susceptibility to ferroptosis. To further confirm the pivotal role of DHFR degradation in increasing ferroptosis susceptibility, we treated A549 cells with methotrexate (MTX), a known inhibitor of DHFR [15], and results showed that MTX treatment significantly increased ferroptosis susceptibility of A549 cells (Fig. 4E and F). Furthermore, we replenished DHFR in CD38-overexpression cells (Fig. S2A and S2B). Results demonstrated that the survival rates increased after DHFR replenishment (Fig. 4G and H). These results indicated that replenishing NMN or DHFR rescued A549-CD38 cells from ferroptosis, thereby further confirming that the oxidative degradation of DHFR was responsible for the increased ferroptosis susceptibility. Since it has been proved that the oxidative degradation of DHFR was dependent on the autophagy and non-canonical proteasome pathways, we further compared the ferroptosis susceptibility of A549-CD38 cells with or without Baf-A1 and MG132 treatment. The results showed that Baf-A1 but not MG132 alleviated the susceptibility to ferroptosis in A549-CD38 cells (Fig. 4I). It has been reported that MG132 treatment alone could decrease cell survival while inhibiting the proteasome activity. According to our results, MG132 alone indeed significantly reduced cell survival rate by about 32% while Baf-A1 had little effect on the cell survival rate of A549-CD38 cells, which may explain why MG132 failed to rescue cells in the case of ferroptosis (Fig. 4J).

CD38 is necessary for immune cell activation and proliferation, such as macrophages, natural killer (NK) cells, regulatory T cells and so on. To further verify the oxidative degradation of DHFR, we overexpressed CD38 in RAW264.7 cells (RAW-CD38 cells), a mouse macrophage cell line (Fig. S3A). ROS levels were increased while DHFR protein levels were decreased in RAW-CD38 cells (Fig. S3B and S3C). Besides, NMN treatment increased the protein levels of DHFR and in the meantime decreased ROS levels in RAW-CD38 cells just as in A549-CD38 cells (Fig. S3C and S3E), during which the transcription levels of DHFR were not significantly changed (Fig. S3D). In addition, NAc, Baf-A1, and MG132 treatment could also increase the DHFR protein levels in RAW-CD38 cells, which further indicated that NAD^+^ decline caused by CD38 high expression induced DHFR oxidative degradation dependent on the autophagy and non-canonical proteasome pathways (Fig. S3F). Moreover, RAW-CD38 cells were more susceptible to Erastin or RSL3-induced ferroptosis, especially RSL3-induced ferroptosis (Fig. S3G and S3H). As in A549-CD38 cells, NMN and Baf-A1, but not MG132, alleviated the ferroptosis susceptibility in RAW-CD38 cells (Fig. S3I). Consistently, MG132 treatment alone decreased the cell survival rate by about 65% while Baf-A1 barely changed the cell survival rate (Fig. S3J), which might account for the failure of MG132 to mitigate ferroptosis.

### BMDMs from aged mice are more susceptible to ferroptosis than those from young mice

Systemic inflammation accompanied by the decline in immune functions is related to age, is newly named “inflammaging” [32, 33]. Recent research revealed that senescence-associated secretory phenotype (SASP) upregulated CD38 expression levels in macrophages [5, 34]. However, it is unknown whether immune cells such as macrophages in aged organisms also increase the susceptibility to ferroptosis, which in turn leads to functional decline. Therefore, we took BMDMs as an example to further explore whether ferroptosis susceptibility is increased during aging due to the high expression of CD38.

**Fig. 5:**
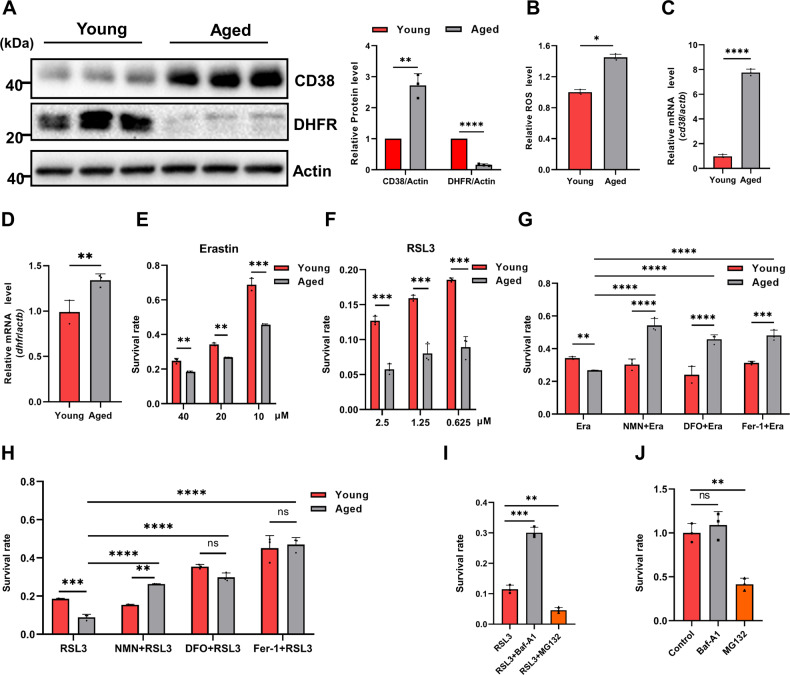
BMDMs from aged mice are more susceptible to ferroptosis. **A** Western blot analysis of CD38, DHFR, and Actin (loading control) in BMDMs from young and aged mice, respectively. Graphs represent the quantification of the blots (*n* = 3). **B** The relative ROS levels of BMDMs from young and aged mice (*n* = 3). **C** and **D** The mRNA expression levels of *cd38* and *dhfr* in BMDMs from young and aged mice, respectively (*n* = 3). **E** and **F** The survival rate of BMDMs from young and aged mice under Erastin (**E**) or RSL3 (**F**) treatment (*n* = 3). **G** The survival rate of BMDMs from young and aged mice under Erastin (20 μM), Erastin (20 μM) + NMN (1 mM), Erastin (20 μM) + DFO (50 μM), Erastin (20 μM) + Fer-1 (1 μM) treatment for 12 h (*n* = 3). **H** The survival rate of BMDMs from young and aged mice under RSL3 (0.625 μM) or + RSL3 (0.625 μM) + NMN (1 mM), RSL3 (0.625 μM) + DFO (50 μM), RSL3 (0.625 μM) + Fer-1 (1 μM) treatment for 12 h (*n* = 3). **I** The survival rate of BMDMs from aged mice under RSL3 (0.625 μM, 12 h), RSL3 (0.625 μM) + Baf-A1 (100 nM, co-treatment with RSL3 for 12 h), and RSL3 (0.625 μM) + MG132 (10 μM, pre-treatment for 4 h) (*n* = 3). **J** The survival rate of BMDMs from aged mice under DMSO (Control), Baf-A1 (100 nM), and MG132 (10 μM, pre-treatment for 4 h) treatment for 12 h (*n* = 3). Data were shown as mean ± SD and analyzed by Student’s *t*-test or one-way ANOVA test. *p < 0.05, **p < 0.01, ***p < 0.001, ****p < 0.0001.

As reported, the protein and mRNA levels of CD38 in BMDMs from aged mice were indeed increased compared to those from young mice (Fig. 5A and C). Besides, ROS levels were increased while the protein levels of DHFR were decreased in BMDMs from aged mice (Fig. 5A and B). Consistent with the results of A549-CD38 cells and RAW-CD38 cells, the transcription levels of DHFR were not decreased in BMDMs from aged mice compared to those from young mice (Fig. 5D). Therefore, we speculated that the decrease of DHFR in BMDMs from aged mice likely resulted from oxidative degradation due to the increased oxidative stress induced by high expression of CD38.

As expected, BMDMs from aged mice were more susceptible to ferroptosis (Fig. 5E and F). NMN could significantly alleviate ferroptosis induced by Erastin or RSL3 in BMDMs from aged mice, as ferroptosis inhibitors DFO and Fer-1 (Fig. 5G and H). Moreover, Baf-A1 but not MG132 was able to alleviate ferroptosis susceptibility of BMDMs from aged mice (Fig. 5I), which may also be due to the reduced cell survival under MG132 treatment (Fig. 5J). In conclusion, we proposed that the elevated CD38 expression in BMDMs from aged mice also led to the oxidative degradation of DHFR, and rendered cells more susceptible to ferroptosis. More importantly, NMN supplementation reduced the susceptibility to ferroptosis in BMDMs from aged mice.

## Discussion

It has been well documented that CD38 was upregulated in organs and macrophages in aged mice, which drives down the cellular NAD^+^ level thus leading to age-associated organ malfunctions [4, 5, 7]. Our previous studies have demonstrated that high expression of CD38 increases oxidative stress and downregulates proteins involved in glycolysis, DNA repair, and antioxidation [35]. Importantly, we have revealed that CD38-mediated NAD^+^ decline triggered oxidative degradation of 15-PGDH via protein sulfonation [19]. However, it is not clear whether sulfonation-induced degradation can be applied to the degradation of other proteins. The first aim of the present study was to examine the DHFR downregulation revealed in our proteomic analysis. Using a cell model in which CD38 overexpression decreased the NAD^+^ levels, we demonstrated that NAD^+^ decline induced oxidative degradation of DHFR. We further revealed that ROS-induced sulfonation of Cys7 in DHFR triggered its degradation, which was dependent on the autophagy and non-canonical proteasome pathways. Mutation of Cys7 to alanine (C7A) prevented DHFR degradation while mutation of Cys7 to aspartic acid (C7D) destabilized DHFR protein. Moreover, NMN replenishment reduced DHFR degradation. We also demonstrated that the oxidative degradation of DHFR, a known ferroptosis suppressor, increased the susceptibility to ferroptosis. Collectively, we showed that NAD^+^ decline caused by CD38 high expression increased cellular susceptibility to ferroptosis via DHFR oxidative degradation, which was reverted by NMN supplementation.

To reveal the physiological relevance of the present finding, we isolated bone marrow monocytes from young and aged mice and induced them into BMDMs to verify the findings from A549-CD38 cells and RAW-CD38 cells. Consistent with the results of A549-CD38 cells and RAW-CD38 cells, BMDMs from aged mice were more susceptible to ferroptosis with higher levels of CD38 and lower levels of DHFR compared with those from young mice. BMDMs from aged mice were indeed more susceptible to ferroptosis. Besides, NMN also alleviated the ferroptosis susceptibility of BMDMs in aged mice. Aging is a multifactorial, inevitable process characterized as a gradual and progressive functional decline of physiological functions [36], imbalance of pro and antioxidants [37], ferrous ion retention, increased oxidative stress, and elevated inflammatory response [17, 38, 39]. Aging also increases the risk of various diseases, such as neurological disorders, diabetes, cancers, and ARDs. Therefore, the characterization of aging-associated ferroptosis in mammalian tissues and organs is important for understanding the aging process [40].

In conclusion, our results demonstrated that oxidative degradation via protein sulfonation can be applied to the downregulation of DHFR in CD38-overexpression cells, and CD38-mediated NAD^+^ decline increased ferroptosis susceptibility via DHFR oxidative degradation. Consistently, BMDMs from aged mice were more susceptible to ferroptosis with higher expression of CD38 and lower expression of DHFR. These results proposed that CD38 upregulation during aging rendered cells more susceptible to ferroptosis. On the other hand, NMN executes its anti-aging function by inhibiting ferroptosis.

## Materials and methods

### Cell culture

Human lung cancer cell line A549 (Male), mouse fibroblast cell line L929 (Male), and human embryonic kidney cell line 293T (Female) were obtained from the cell bank of the Chinese Academy of Sciences (Shanghai, China). The mouse macrophage cell line RAW264.7 was a generous gift from Xin Lin Laboratory, School of Life Sciences, Tsinghua University, Beijing, China. A549 cells were grown in RPMI-1640 medium (Wisent, Montreal, QC) supplemented with 10% FBS (Wisent, Montreal, QC) and 1% penicillin/streptomycin (Wisent, Montreal, QC). L929 cells were grown in RPMI-1640 medium (Wisent, Montreal, QC) supplemented with 10% heat-inactivated FBS (Wisent, Montreal, QC) and 1% penicillin/streptomycin (Wisent, Montreal, QC). 293T cells were grown in Dulbecco’s modified Eagle’s medium (Wisent, Montreal, QC) supplemented with 10% FBS (Wisent, Montreal, QC) and 1% penicillin/streptomycin (Wisent, Montreal, QC). RAW264.7 cells were cultured in Dulbecco’s modified Eagle’s medium (Wisent, Montreal, QC) supplemented with 10% heat-inactivated FBS (Wisent, Montreal, QC) and 1% penicillin/streptomycin (Wisent, Montreal, QC). The mycoplasma contamination test for all cells was negative.

### Cell line construction

The pLVX-CD38-IRES-ZsGreen1 plasmid was constructed previously, on which CD38 DNA sequence encoding 2–43 amino acids were deleted and a Flag-tag was added at its C-terminus. Then the CD38 DNA sequence was obtained from the pLVX-CD38-IRES-ZsGreen1 plasmid and cloned into the pLVX-IRES-mCherry plasmid to construct the pLVX-CD38-IRES-mCherry plasmid for further use. The construction of the pLVX-cd38-IRES-ZsGreen1 plasmid was similar. Besides, the DHFR cDNA sequence was obtained from A549 cells and then cloned into the pLVX-IRES-ZsGreen1 plasmid with a Flag-tag added at its C-terminus hereinafter called pLVX-DHFR-IRES-ZsGreen1.

293T cells were transfected with target plasmids together with helper plasmids for lentivirus packaging. Then A549 cells were infected with lentivirus and sorted by the BD FACSAria II Flow Cytometer (BD Biosciences, NJ, USA) to construct overexpression cell lines.

### Cellular reactive oxygen species (ROS) measurement

The cellular ROS levels were determined by CellROX Deep Red Reagent (Invitrogen, NY, USA). Briefly, CellROX Deep Red reagent was applied to cells in a complete medium and incubated for 30–60 min in the cell incubator. Then cells were analyzed for the mean fluorescence intensity at 665 nm by the BD FACSAria II Flow Cytometer (BD Biosciences, NJ, USA).

### Western blotting

Cells were lysed in RIPA lysis buffer (Bryotime, Shanghai, China) supplemented with 1× protease inhibitor cocktail (Thermo-Pierce Biotechnology, Rockford, IL) and sonicated on ice. The protein concentration was determined by the BCA protein concentration assay kit (Bryotime, Shanghai, China) and equal amounts of proteins were separated by gel electrophoresis, and then electroblotted to the polyvinylidene difluoride (PVDF) membrane, blocked with 5% non-fat milk, incubated with the primary antibody and the secondary antibody sequentially. Finally, target proteins were detected using ECL reagent (YESEN, Shanghai, China) and photographed by ChemiDoc^TM^ XRS+ SYSTEM (Bio-Rad, CA, USA). Primary antibodies for β-Actin (ABclonal, Woburn, MA), DHFR (Abcam, Cambridge, UK), CD38-human, CD38-mouse, Flag (Cell Signaling Technology, Danvers, MA), and secondary anti-rabbit HRP-IgG antibodies (Cell Signaling Technology, Danvers, MA) were used for detecting target proteins.

### RNA isolation and real-time quantitative polymerase chain reaction analysis (RT-qPCR)

Total RNA was extracted by TRNzol Universal Total RNA Extraction Reagent (TIANGEN, Beijing, China), which was then reversely transcribed into cDNA using reverse transcriptase (CWBIO, Beijing, China). SYBR green reaction mixture (CWBIO, Beijing, China) was used to perform qPCR with the Roche LightCycler 96 System (Roche, Basel, Switzerland). And *ACTB* was used as an internal control. All samples were performed in triplicate and the data were analyzed by Student’s *t*-test. Primers used in qPCR were listed in Table S1.

### Survival rate measurement

Cell counting kit-8 (CCK-8) was used to determine the survival rate by measuring the absorbance at 450 nm (*A*_450_). Briefly, an equal number of cells were seeded into 96-well plates beforehand and then treated under different conditions. After that, cells were washed with PBS and a complete medium supplemented with 10% CCK-8 reagent (APExBIO, Boston, MA) was added to each well including the blank well (Blank control group). Then the plates were incubated in the cell incubator for about 2 h for further measuring *A*_450_. The survival rate was calculated according to the formula survival rate = (*A*_450 Treatment group_ − *A*_450 Blank control group_)/(*A*_450 Control group_ − *A*_450 Blank control group_). All the experiments were performed in triplicate and the data were analyzed by Student’s *t*-test or one-way ANOVA test.

### Immunoprecipitation

For identifying the oxidative modification of DHFR, equal amounts of proteins from untreated, and 1 mM H_2_O_2_ + MG132-treated A549-DHFR cells were incubated with Anti-Flag Affinity Gel (Bimake, Houston, TX, USA) at 4 °C on the rotator for about 6 h. After washing beads five times, 200 μL 0.1 M Glycine HCl (PH = 3) was applied to the beads and then rotated at a low speed for 5–10 min in order to elute the enriched DHFR-Flag protein. After that, the supernatant was adjusted to around PH = 7.4 and then added with 5× non-reducing SDS–PAGE loading buffer (CWBIO, Beijing, China) followed by in-gel digestion and LC–MS/MS analysis.

### In-gel digestion followed by LC–MS/MS analysis

Protein samples were separated by SDS–PAGE electrophoresis and target protein bands were excised, and then alkylated by incubating with 25 mM chloroacetamide for 45 min in the dark at 55 °C followed by digesting using trypsin (Promega, Fitchburg, WI) for 14 h at 37 °C. The peptides were extracted three times with 50% acetonitrile supplemented with 0.1% formic acid and then vacuum-dried peptides were dissolved in 20 µl 0.1% (v/v) formic acid and analyzed by LC–MS/MS analysis.

For LC–MS/MS analysis, peptides were loaded in a trap column and then separated by the Thermo-Dionex Ultimate 3000 HPLC system (Thermo Fisher Scientific, Waltham, MA, USA), and detected by the Obitrap Fusion LUMOS Tribrid mass spectrometer (Thermo Fisher Scientific, Waltham, MA, USA). Identification of protein modification was performed by Proteome Discoverer 2.3 software using the label-free quantification method.

### Metabolomics analysis

The metabolomics analysis was performed as previously mentioned [41]. Briefly, cells were incubated with pre-chilled 80% methanol for 2 h at −80 °C after being washed with PBS three times. Then cells were scraped and centrifuged at 12,000 rpm for 20 min at 4 °C. The supernatant was vacuum dried, redissolved in 80% methanol, and analyzed by the TSQ Quantiva™ triple quadrupole mass spectrometer (Thermo Fisher Scientific, Waltham, MA, USA) with Ultimate 3000 in positive and negative ions switching mode. And the identification and quantification of target metabolites were performed by the Q-exactive mass spectrometer (Thermo Fisher Scientific, Waltham, MA, USA) and TraceFinder according to the retention time and molecular mass of metabolites.

### Isolation and culture of bone-marrow-derived macrophages (BMDMs)

Female C57BL/6J mice were purchased from Jackson Laboratory through Laboratory Animal Research Center. All mice were housed in isolated ventilated cages (maximum five mice per cage) in a Biohazard barrier facility at Tsinghua University. The mice were maintained on a 12/12-h light/dark cycle, 22–26 °C with ad libitum access to diet and tap water under specific pathogen-free conditions. The laboratory animal facility has been accredited by AAALAC and the IACUC of Tsinghua University approved all animal protocols used in this study. Three mice were randomly selected from the young and aged groups (young: 8-week-old female mice; aged: 88-week-old female mice) and sacrificed in accordance with Tsinghua Institutional Animal Care and Use Committee guidelines for animal welfare.

BMDMs were derived from bone marrow monocytes in the femurs of euthanized mice. Briefly, the muscle and connective tissue around the femur were removed. The isolated femurs were washed with 75% ethanol twice, followed by three washes with PBS supplemented with 1% penicillin/streptomycin. The bone marrow was flushed using a syringe, filtered through a 70 μm filter, and centrifuged at 1000 rpm for 10 min at 4 °C. The cell pellet was suspended in BMDM culture medium (RPMI-1640 medium supplemented with 30% L929 medium supernatant, 10% heat-inactivated FBS, and 1% penicillin/streptomycin) and then plated in a 10-cm dish. After culturing for 7 days, more than 95% of the cells were macrophages [5].

### Statistical analysis

All data are expressed as the means ± SD for *n* independent experiments, as indicated in the figure legends (normally, *n* = 3). All statistical analysis was processed using GraphPad Prism (version 8.0). Student’s *t*-test was used to calculate the difference between two independent groups with *F* test followed by corrections. One-way ANOVA test was used to compare three or more independent groups with Brown–Forsythe test and Bartlett’s test and an estimate of variation within each group of data. *p*-value < 0.05 was considered as evidence of statistical significance (*p < 0.05; **p < 0.01; ***p < 0.001, ****p < 0.0001). No statistical methods were used to predetermine the sample size. No blinding method was used for the isolation of BMDMs. There were no animal exclusion criteria.

## Supplementary information

Supplementary information

Original western blots

Reproducibility checklist

## Data Availability

The experimental data in the current study are available from the corresponding author upon reasonable request. No applicable resources were generated during the current study.
