# Supplementary material for: Oxidative degradation of dihydrofolate reductase increases CD38-mediated ferroptosis susceptibility
Source: Cell Death Dis. 2022 Nov 9;13(11):944. doi: 10.1038/s41419-022-05383-7 (PMC9646779; doi:10.1038/s41419-022-05383-7)
Supplement: Supplementary file 1 — Supplementary information [file 41419_2022_5383_MOESM1_ESM.docx]

**Supplementary Information**

**Oxidative Degradation of Dihydrofolate Reductase Increases**

**CD38-Mediated Ferroptosis Susceptibility**

Yingying Ma ^1^, Meiqi Yi ^2^, Weixuan Wang ^3^, Xiaohui Liu ^1^, Qingtao Wang ^4^,

Chongdong Liu ^4^, Yuling Chen ^1,*^, Haiteng Deng ^1,*^

^1^ MOE Key Laboratory of Bioinformatics, Center for Synthetic and Systematic Biology, School of Life Sciences, Tsinghua University, Beijing, 100084, China

^2^ BeiGene (Beijing) Co., Ltd., Beijing, 100084, China

^3^ Institute of Chinese Medicine, Guangdong Pharmaceutical University, Guangzhou, 510006, China

^4^ Beijing Chao-yang Hospital, Capital Medical University, Beijing 100043, China

^*^ Corresponding author

**CORRESPONDING AUTHOR:**

Pro. Haiteng Deng, MOE Key Laboratory of Bioinformatics, Center for Synthetic and Systematic Biology, School of Life Sciences, Tsinghua University, Tel: 86-10-62790498. Address: Biomedical Building, Tsinghua University, Beijing, 100084, China. E-mail: dht@mail.tsinghua.edu.cn.

Dr. Yuling Chen, School of Life Sciences, Tsinghua University, Tel: 86-10-62797838. Address: Biomedical Building, Tsinghua University, Beijing, 100084, China. E-mail: chenyuling2016@mail.tsinghua.edu.cn.

Table of Contents

Supplemental Figure

Fig. S1 DHFR or mutated DHFR protein is overexpressed.

Fig. S2 DHFR is replenished on the basis of CD38 high expression.

Fig. S3 High expression of CD38 in RAW264.7 cells induces DHFR oxidative degradation and increases susceptibility to ferroptosis.

Supplemental Tables

Table S1 Primer sequences for qPCR.

**
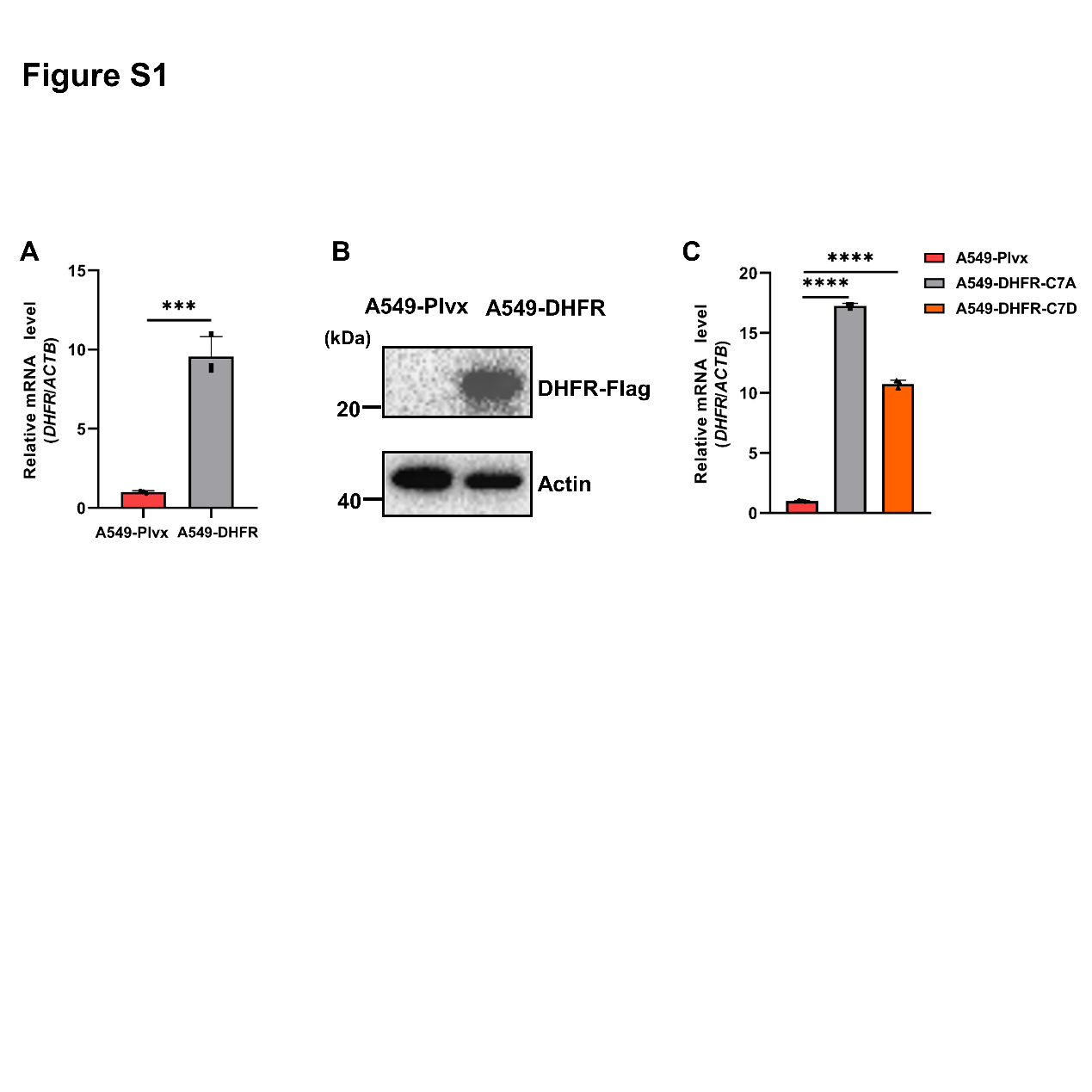
**

Fig. S1 DHFR or mutated DHFR protein is overexpressed. A The relative transcription levels of *DHFR* in A549-Plvx and A549-DHFR cells (n=3). B Western blot analysis of the protein levels of Flag-tagged DHFR in A549-Plvx and A549-DHFR cells. C The relative transcription levels of DHFR in A549-Plvx, A549-DHFR-C7A and A549-DHFR-C7D cells (n=3). Data were shown as mean ± SD and analyzed by Student’s t-test, ***p < 0.001, ****p < 0.0001.


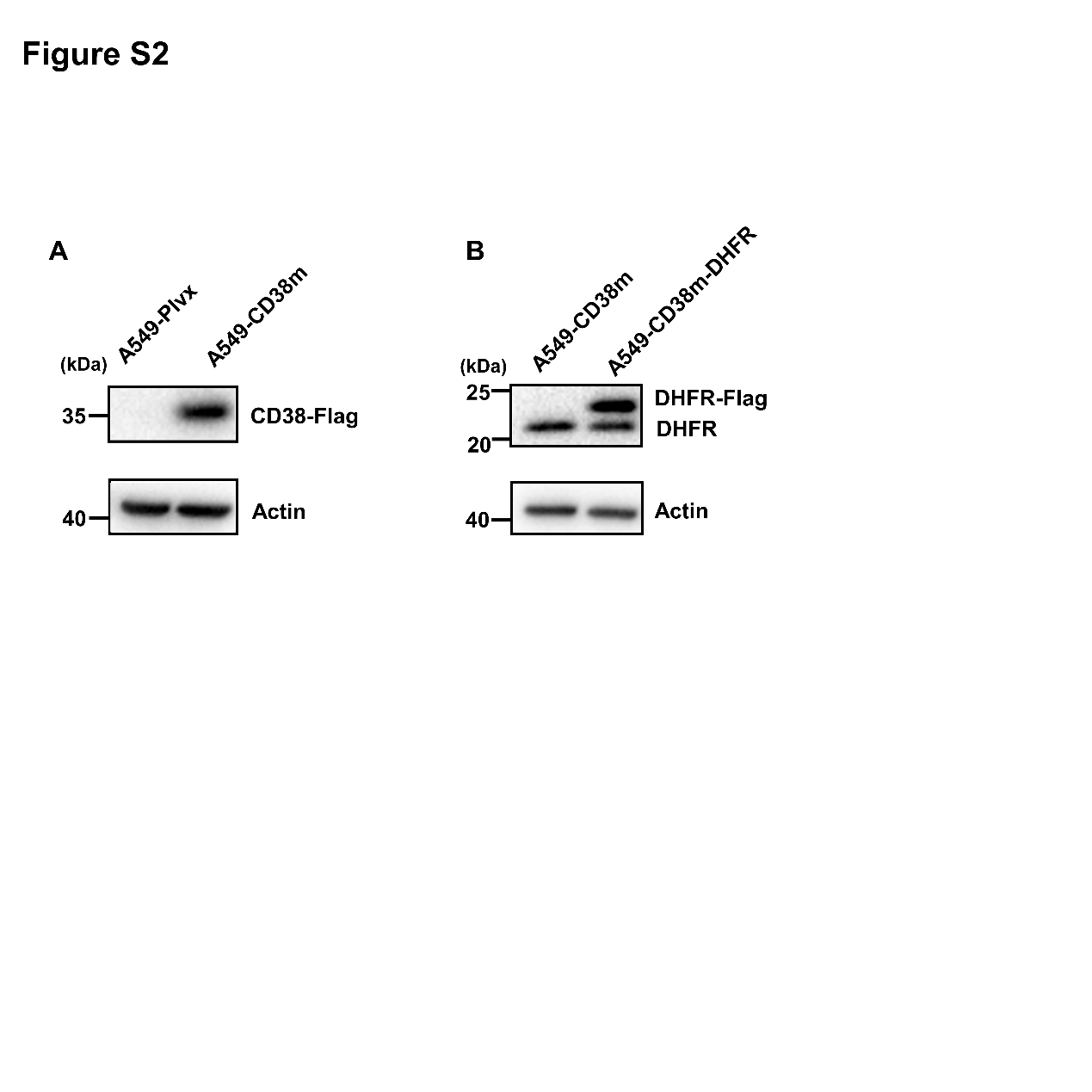


Fig. S2 DHFR is replenished on the basis of CD38 high expression. A Western blot analysis of the protein levels of Flag-tagged CD38 and Actin (loading control) in A549-Plvx and A549-CD38m cells. B Western blot analysis of the protein levels of DHFR, Flag-tagged DHFR and Actin (loading control) in A549-CD38m cells and A549-CD38m-DHFR cells.


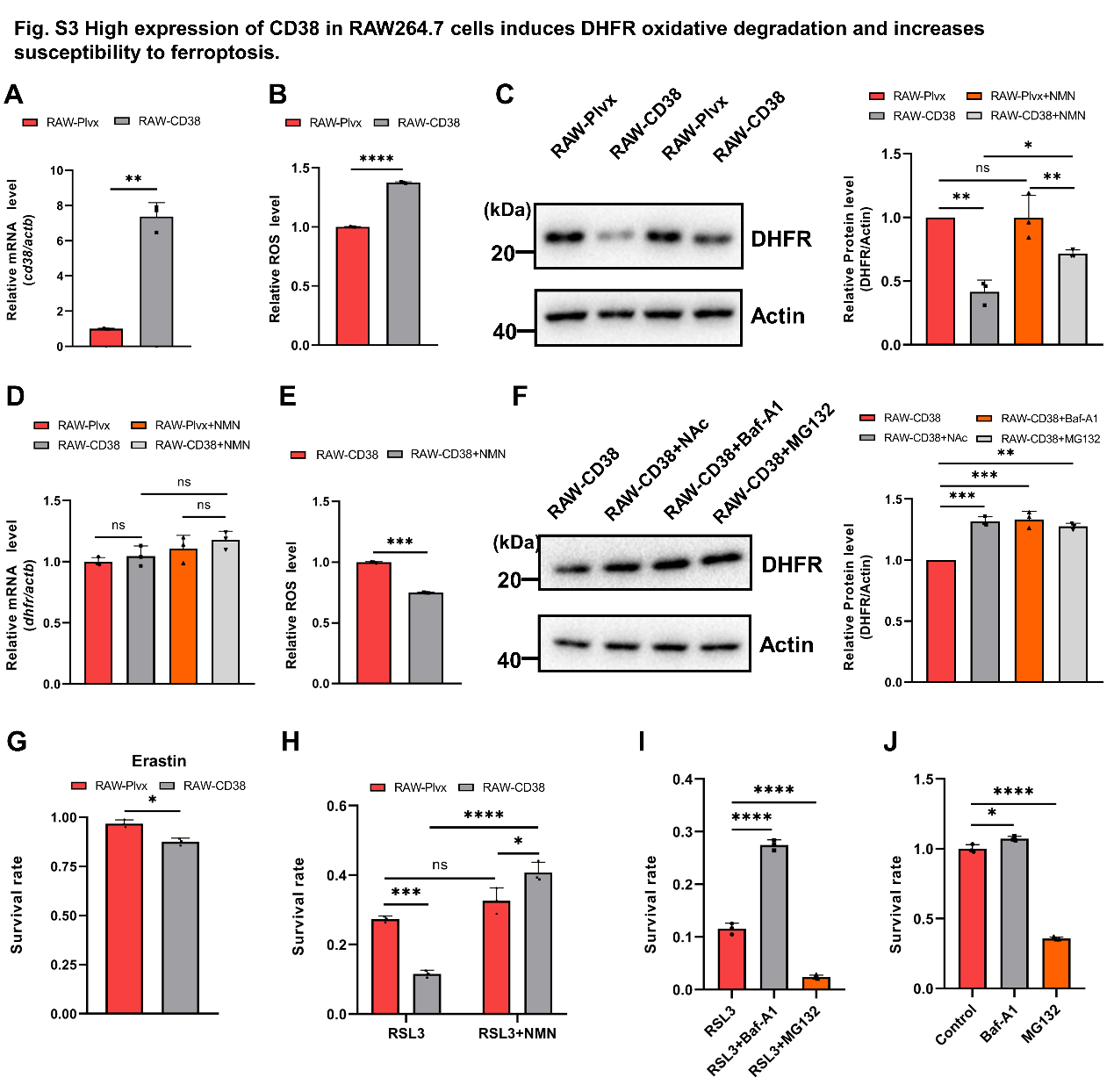


Fig. S3 High expression of CD38 in RAW264.7 cells induces DHFR oxidative degradation and increases susceptibility to ferroptosis. A The relative transcription levels of *cd38* in RAW-Plvx and RAW-CD38 cells (n=3). B Relative ROS levels of RAW-Plvx and RAW-CD38 cells (n=3). C RAW-Plvx and RAW-CD38 cells were treated with or without 1 mM NMN. Protein levels of DHFR and Actin (loading control) were analyzed by western blot. Graphs represent the quantification of the blots (n=3). D The relative transcription levels of *dhfr* in RAW-Plvx and RAW-CD38 cells treated with or without 1 mM NMN (n=3). E Relative ROS levels of RAW-CD38 cells treated with or without 1 mM NMN (n=3). F Western blot images of DHFR and Actin (loading control) in DMSO, NAc (6 mM, 6 h), Baf-A1 (100 nM, 6 h), and MG132 (10 μM, 4 h)-treated RAW-CD38 cells. Graphs represent the quantification of the blots (n=3). G-H The survival rate of RAW-Plvx and RAW-CD38 cells treated with Erastin (40 μM) or RSL3 (5 μM) and RSL3 (5 μM) + NMN (1 mM) for 12 h (n=3). I The survival rate of RAW-CD38 cells under RSL3 (5 μM, 12 h), RSL3 (5 μM) + Baf-A1 (100 nM, co-treatment with RSL3 for 12 h), and RSL3 (5 μM) + MG132 (10 μM, pre-treatment for 4 h) (n=3). J The survival rate of RAW-CD38 cells under DMSO (Control), Baf-A1 (100 nM), and MG132 (10 μM, pre-treatment for 4 h) treatment for 12 h (n=3). Data were shown as mean ± SD and analyzed by Student’s t-test or one-way ANOVA test. *p < 0.05, **p < 0.01, ***p < 0.001, ****p < 0.0001.

Table S1 Primer sequences for qPCR.

| **Genes** | **Species** | **Forward Primer (5'-3')** | **Reverse Primer (5'-3')** |
| --- | --- | --- | --- |
| *CD38* | Human | TGCCAAAGTGTATGGGATGC | TGCAGTCCTTTCTCCAGTCT |
| *DHFR* | Human | CAGCAGAGAACTCAAGGAAC | CCAACTATCCAGACCATGTC |
| *ACTB* | Human | CATGTACGTTGCTATCCAGGC | CTCCTTAATGTCACGCACGAT |
| *cd38* | Mouse | TCTCTAGGAAAGCCCAGATCG | AGAAAAGTGCTTCGTGGTAGG |
| *dhfr* | Mouse | CCTGGTTCTCCATTCCTGAG | GCCTGGGTATTCTGGGAGA |
| *actb* | Mouse | GATCTGGCACCACACCTTCT | GGGGTGTTGAAGGTCTCAAA |
