# Supplementary material for: Oxidative degradation of dihydrofolate reductase increases CD38-mediated ferroptosis susceptibility
Source: Cell Death Dis. 2022 Nov 9;13(11):944. doi: 10.1038/s41419-022-05383-7 (PMC9646779; doi:10.1038/s41419-022-05383-7)
Supplement: Supplementary file 2 — Original western blots [file 41419_2022_5383_MOESM2_ESM.pdf]

Original Western Blots

Fig. 1A   Fig. S2A   Fig. 1F

CD38-Flag

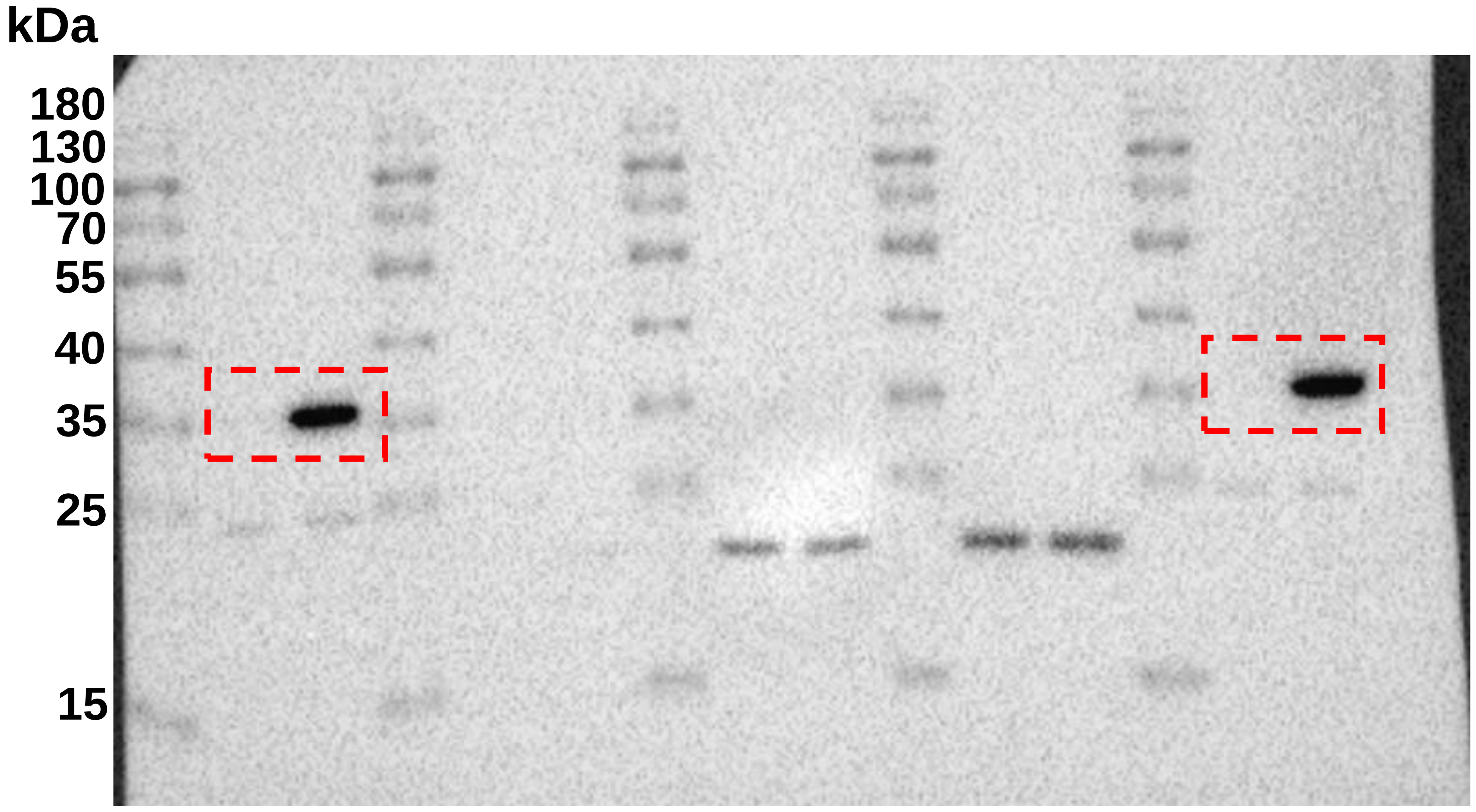

DHFR

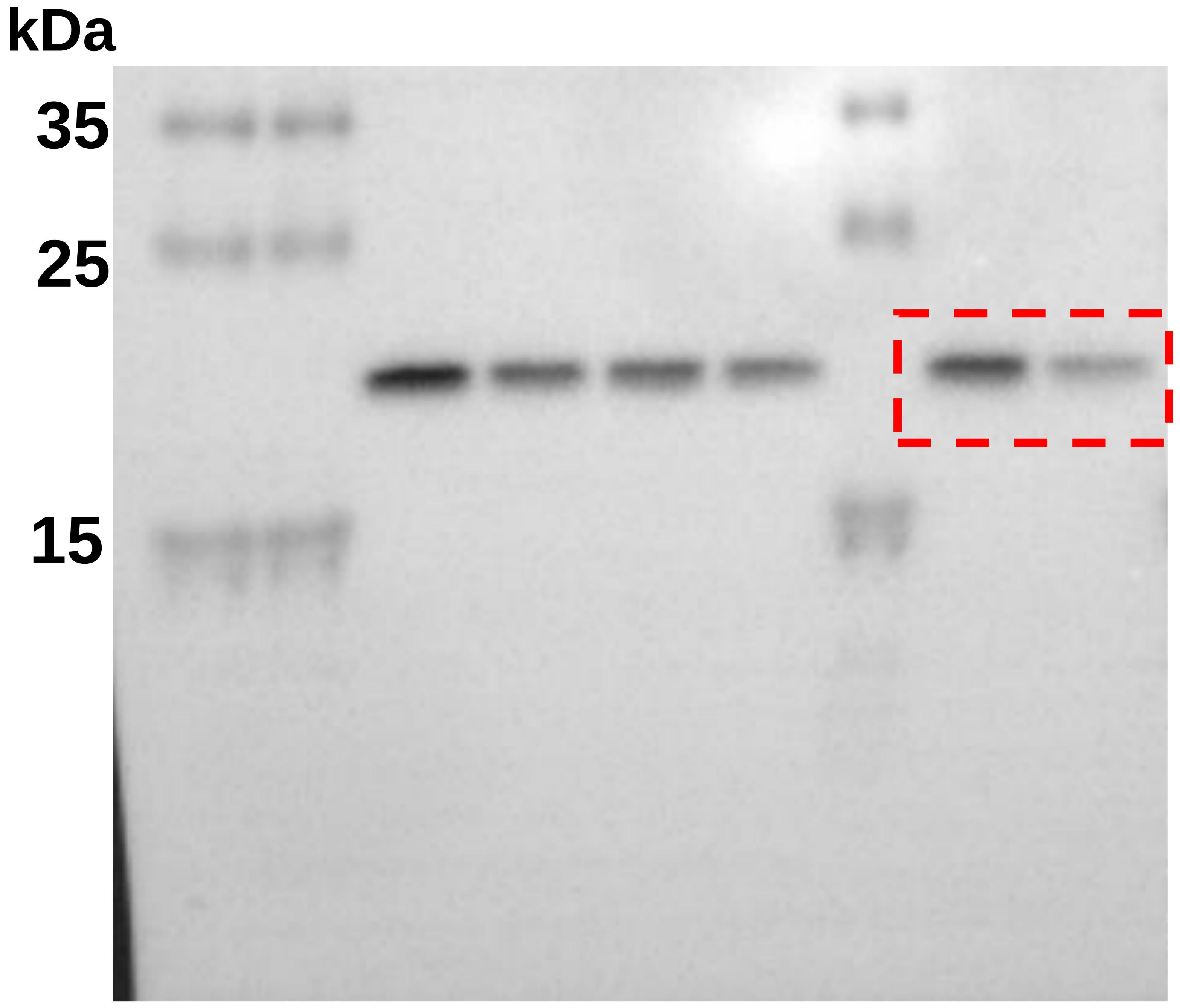

Actin

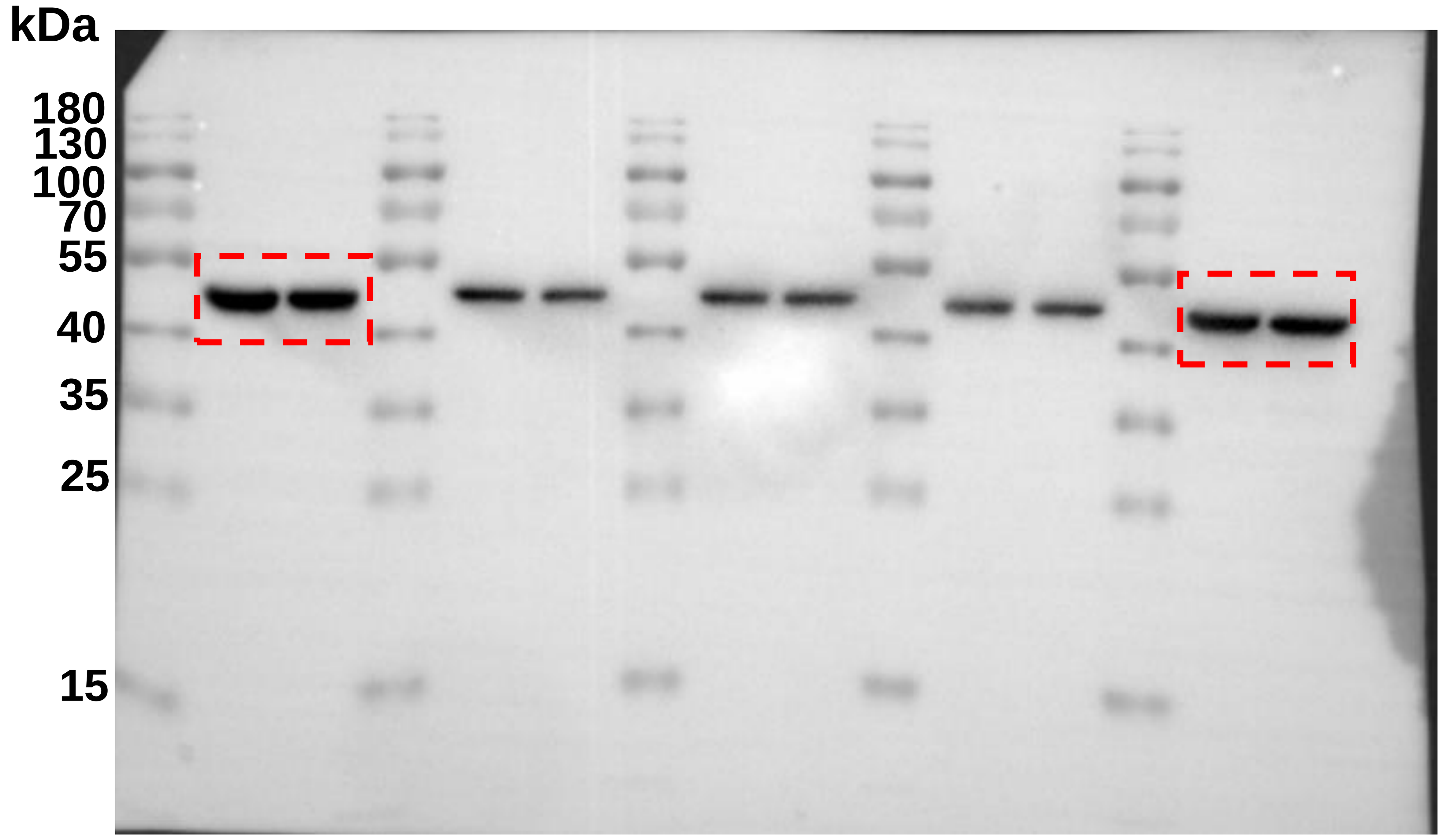

Actin

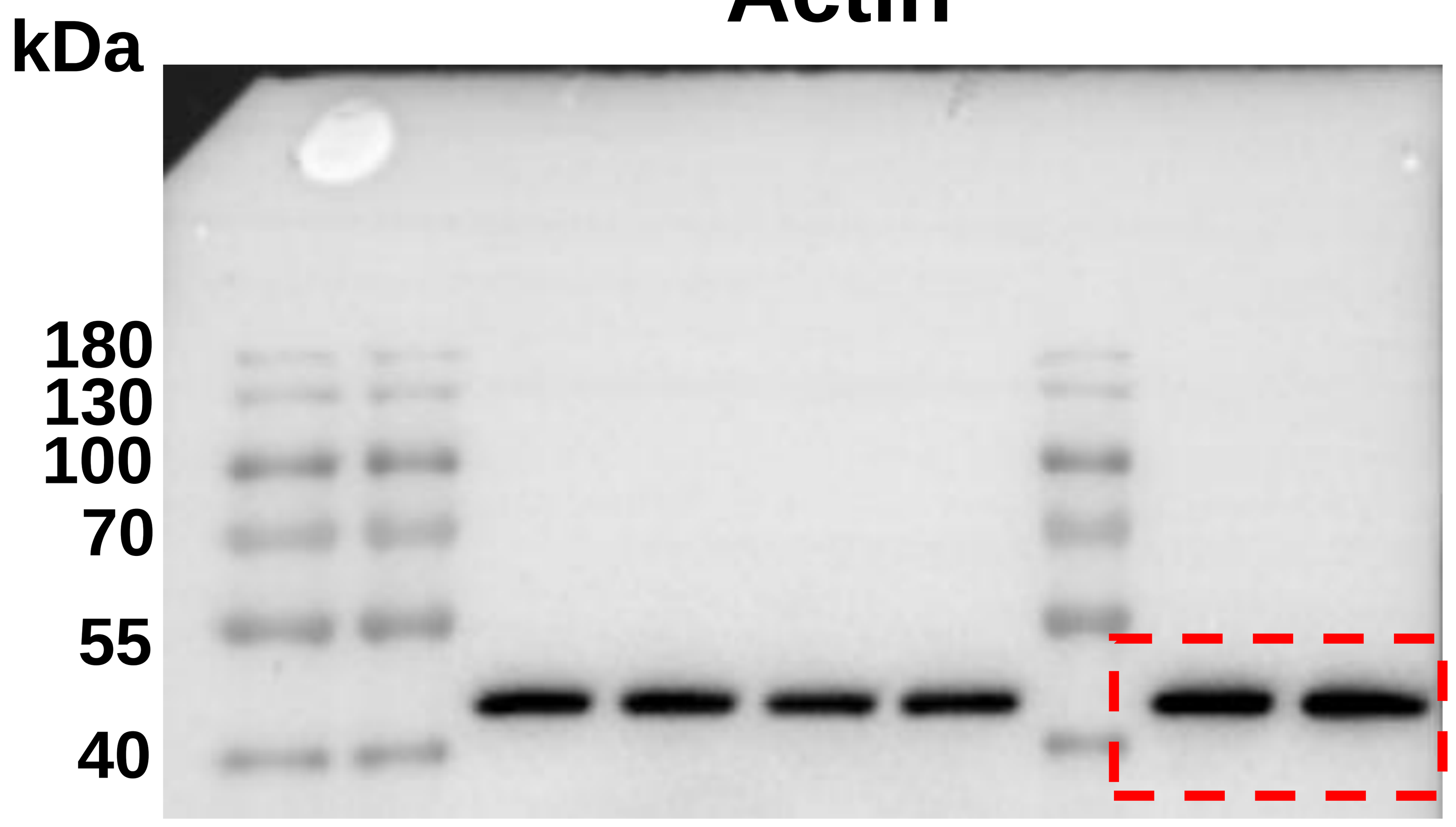

Fig. 1I

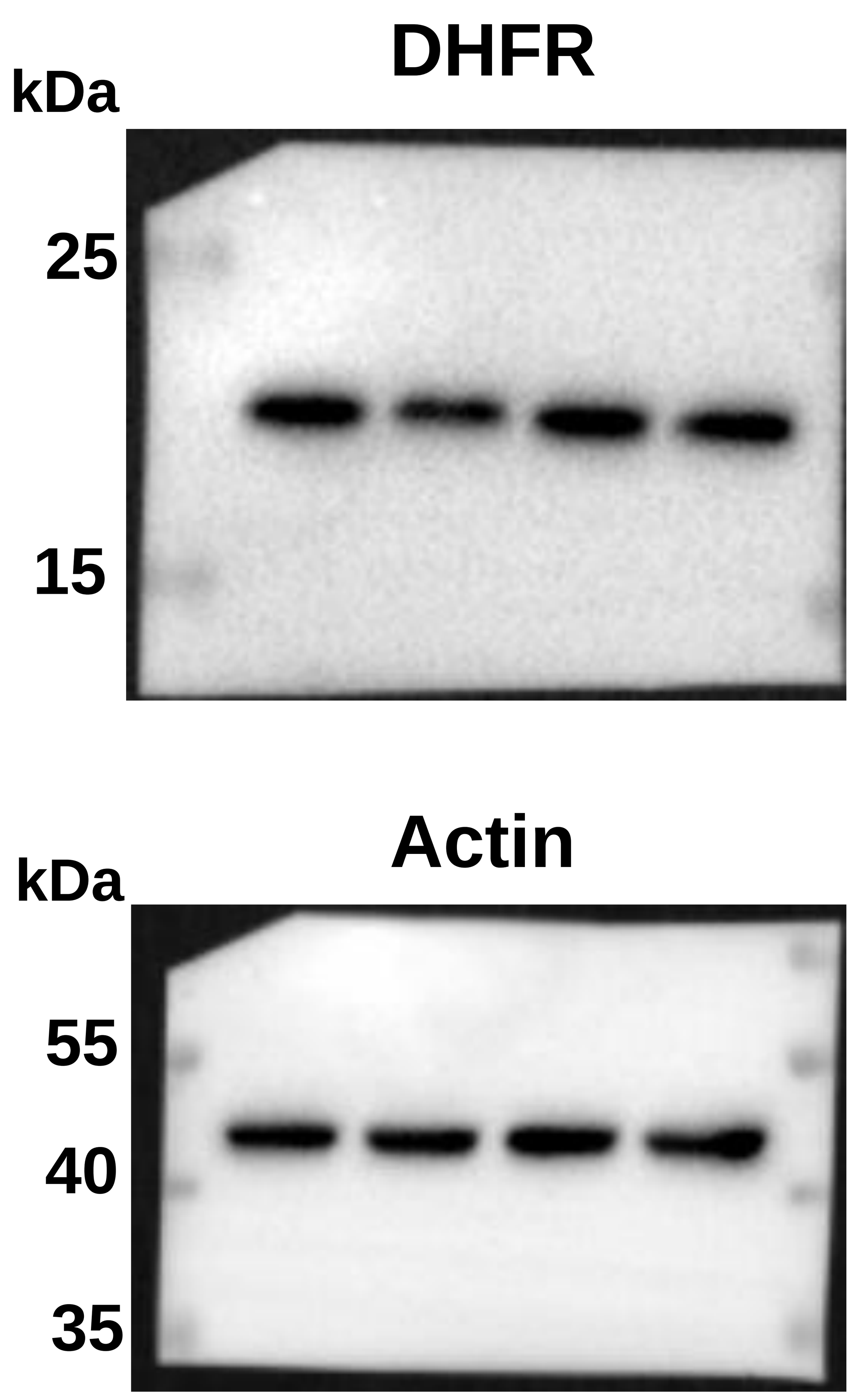
DHFR
kDa
25
15
Actin
kDa
55
40
35

Fig. 2B

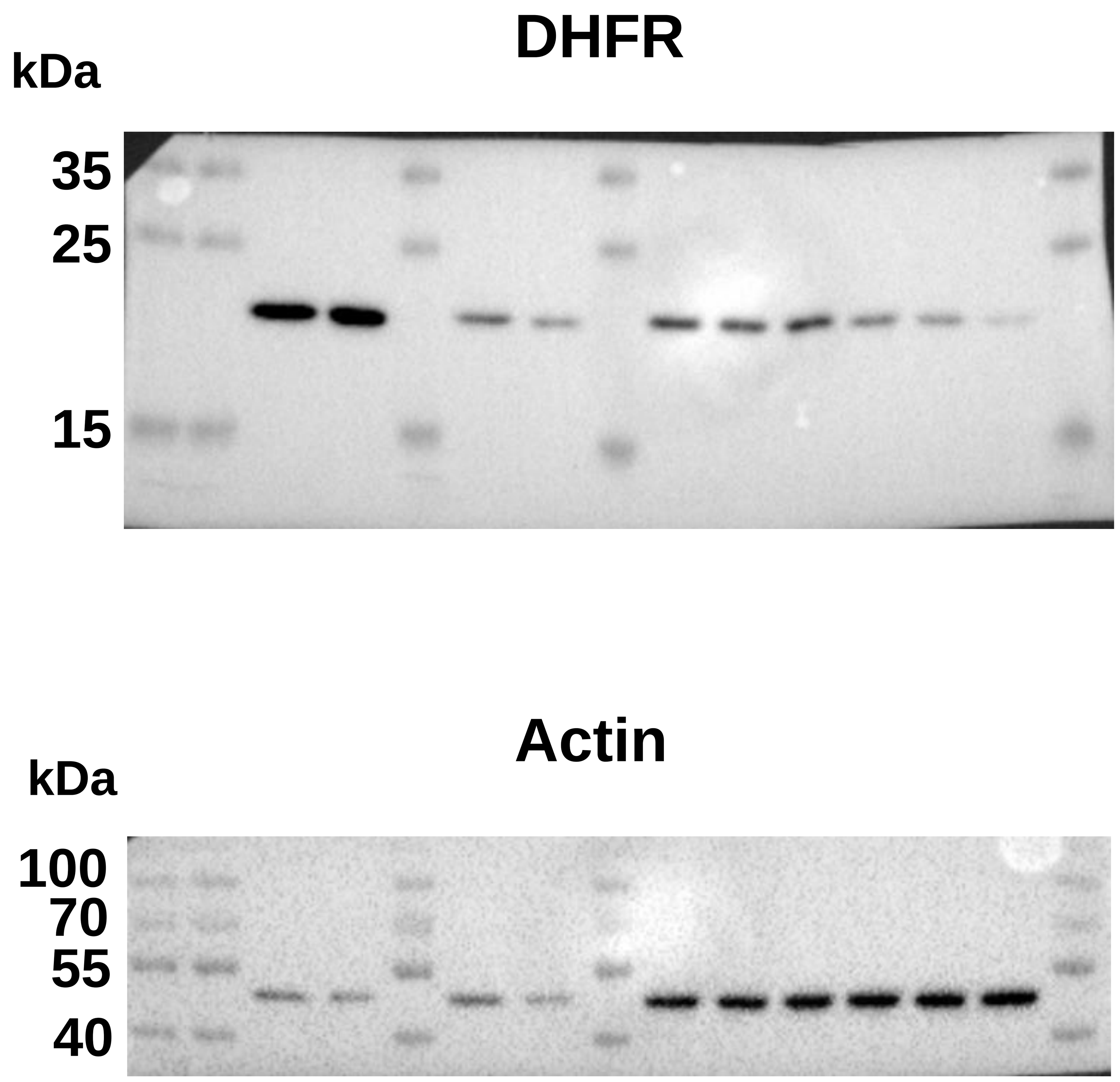
DHFR
kDa
35
25
15
Actin
kDa
100
70
55
40

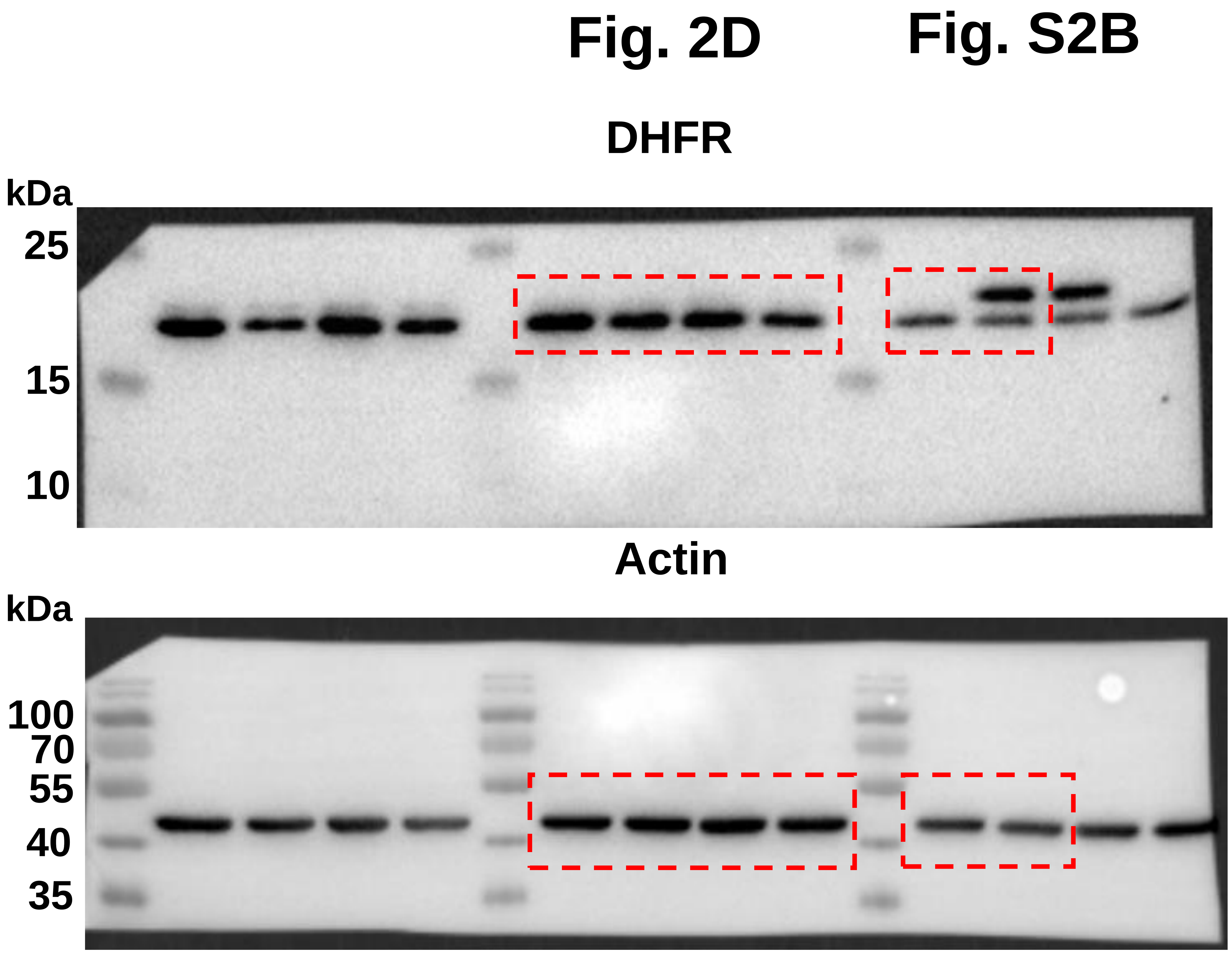

Fig. 2D
Fig. S2B
DHFR
kDa
25
15
10
Actin
kDa
100
70
55
40
35

Fig. 2G Fig. 2E

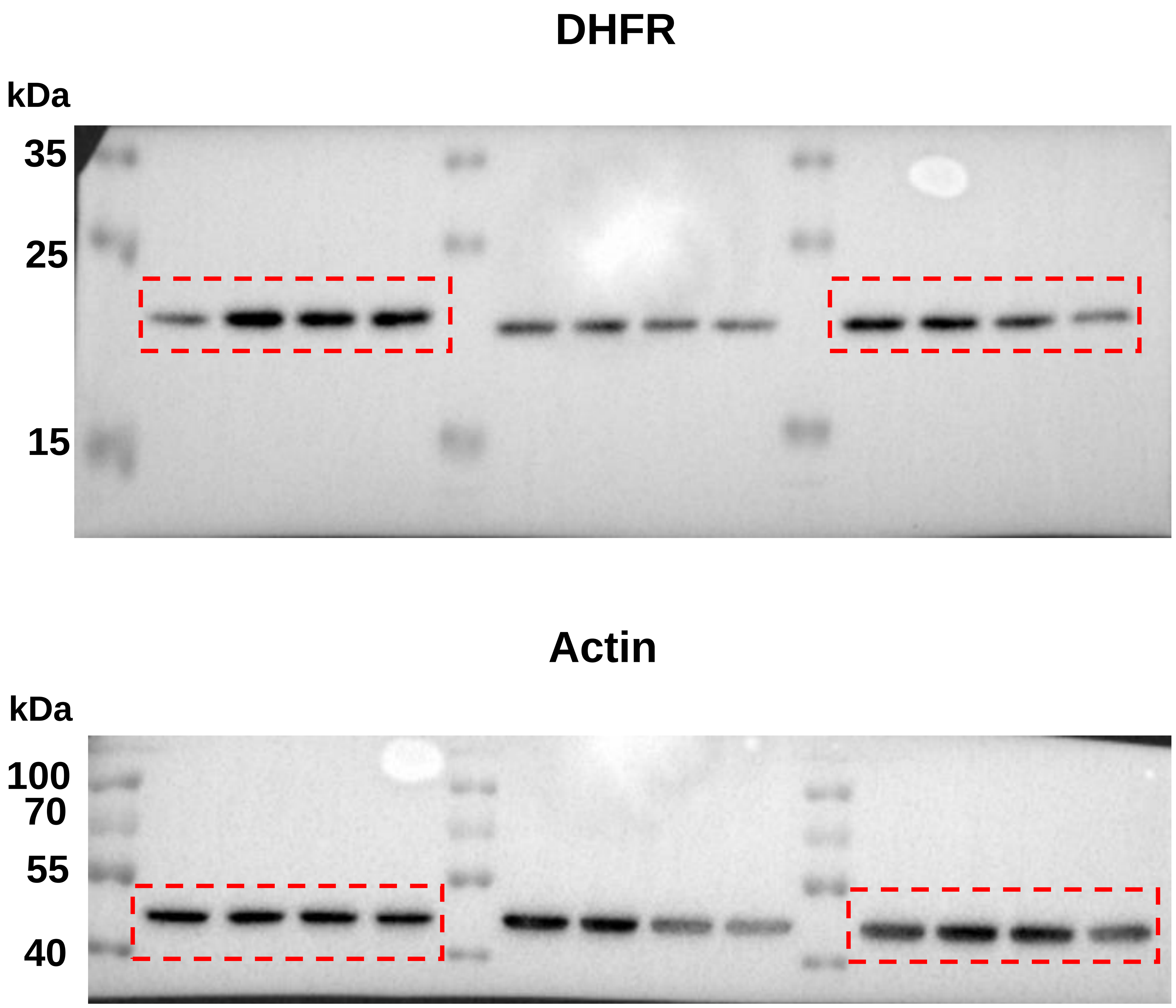

Fig. 2F

DHFR

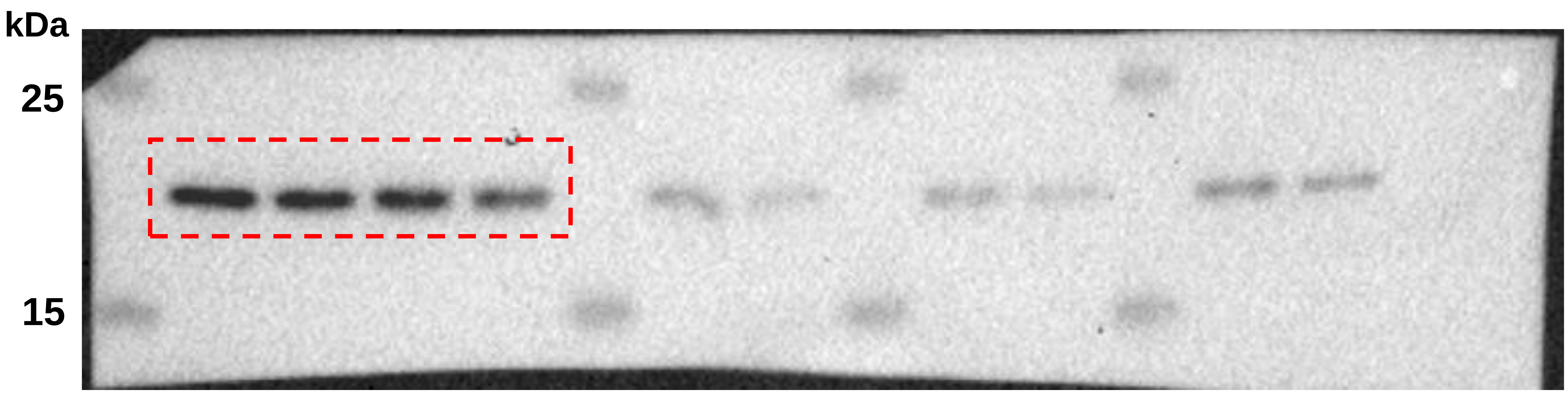

Actin

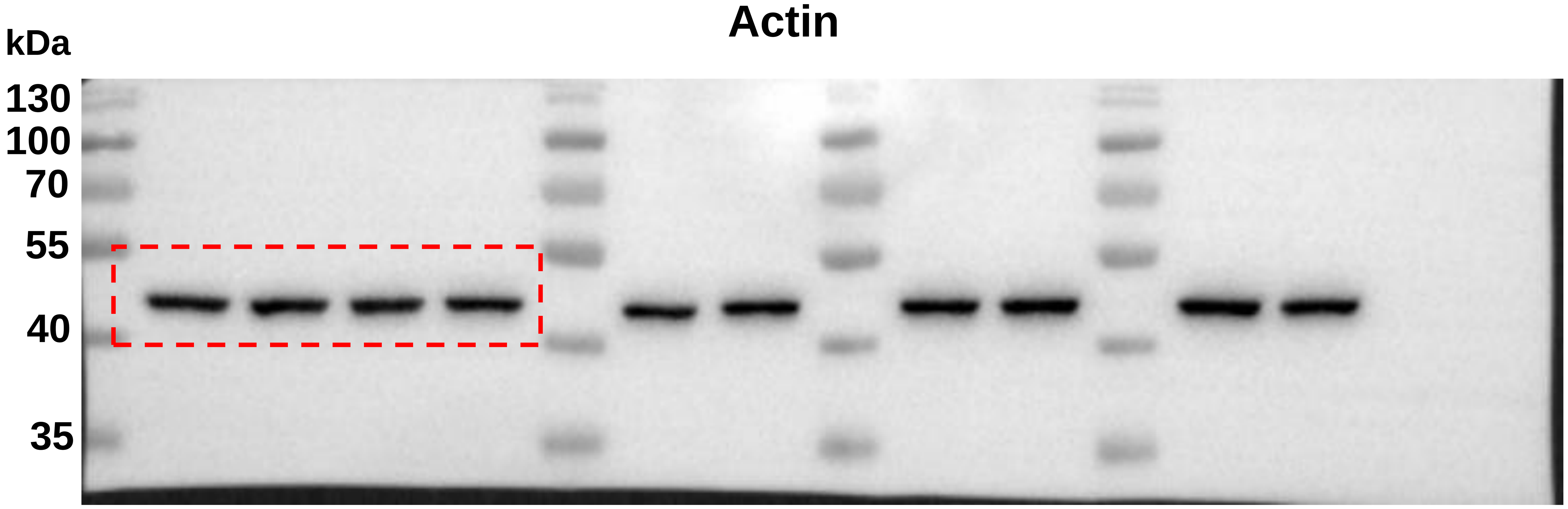

Fig. S1B

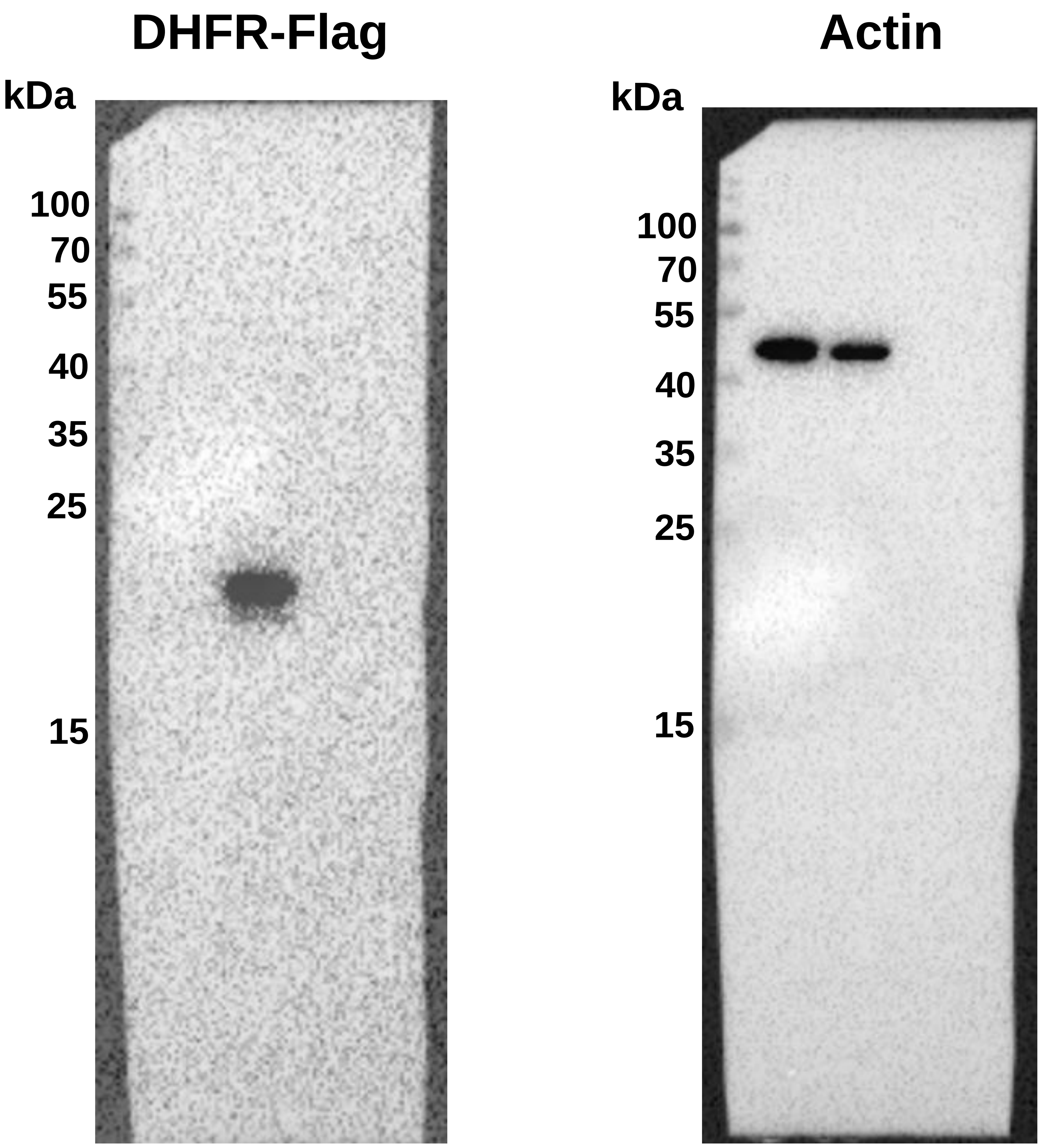

Fig. 3B

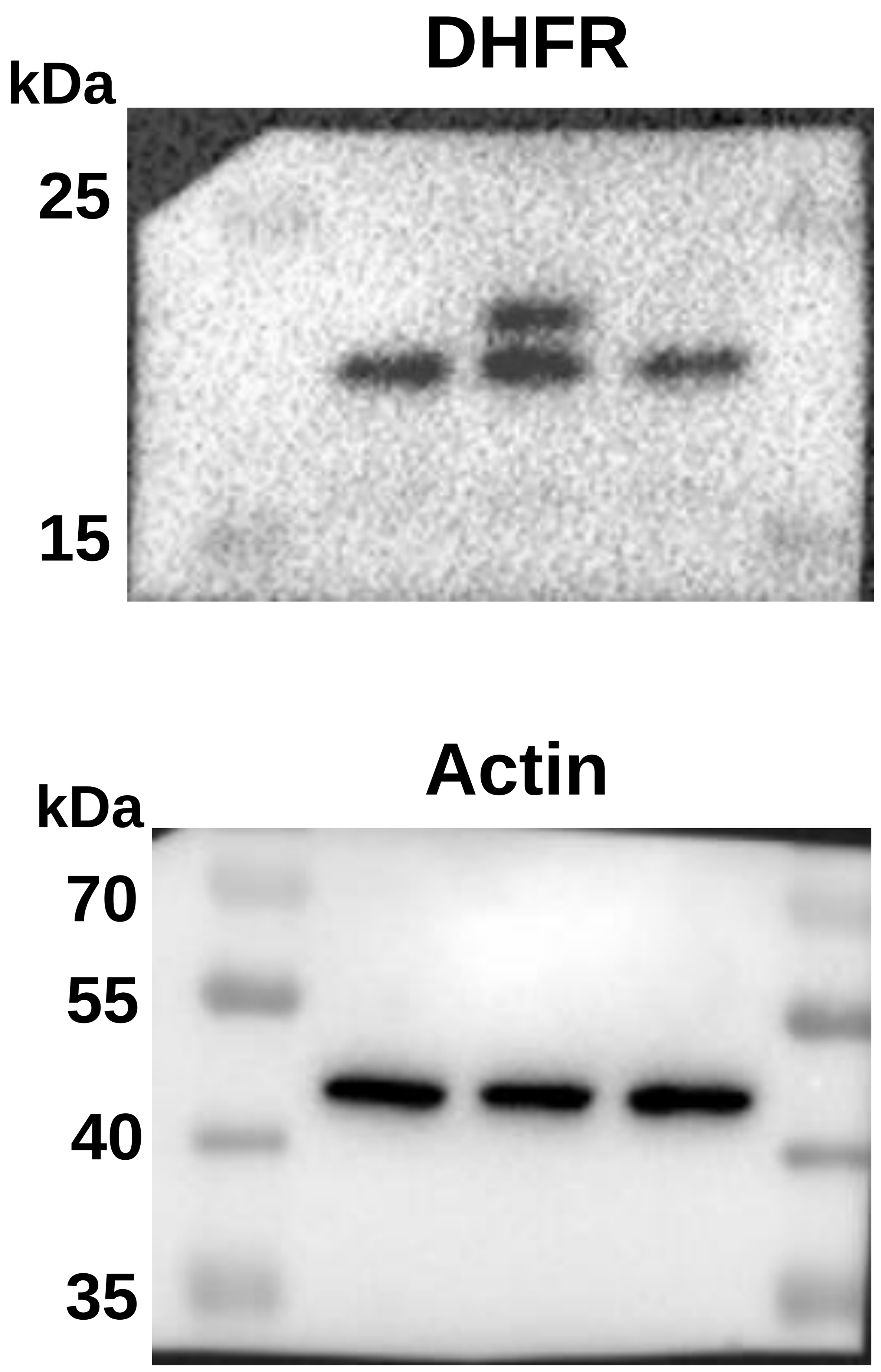
DHFR
kDa
25
15
Actin
kDa
70
55
40
35

Fig. 3C

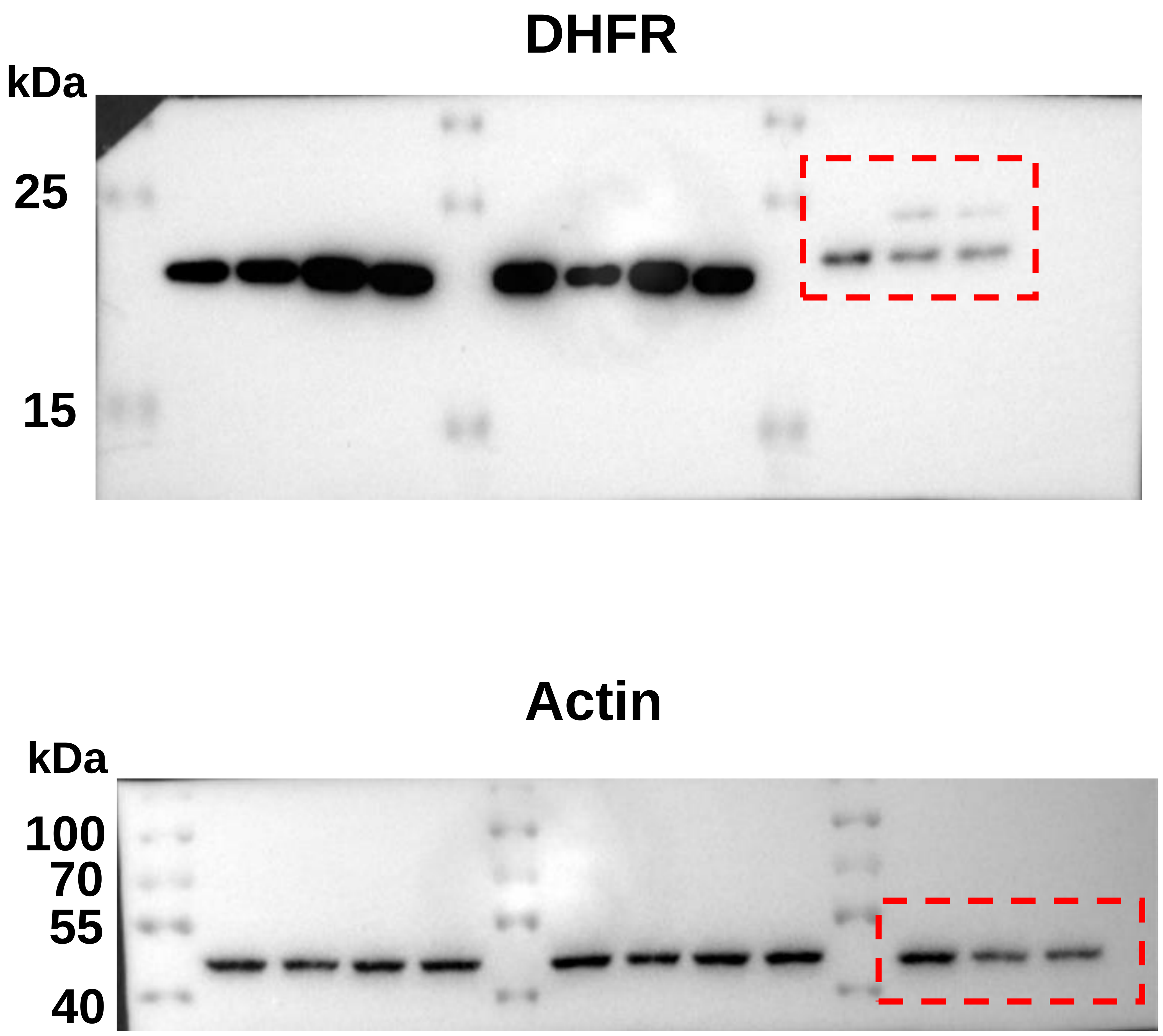
DHFR
kDa
25
15
Actin
kDa
100
70
55
40

Fig. 3D

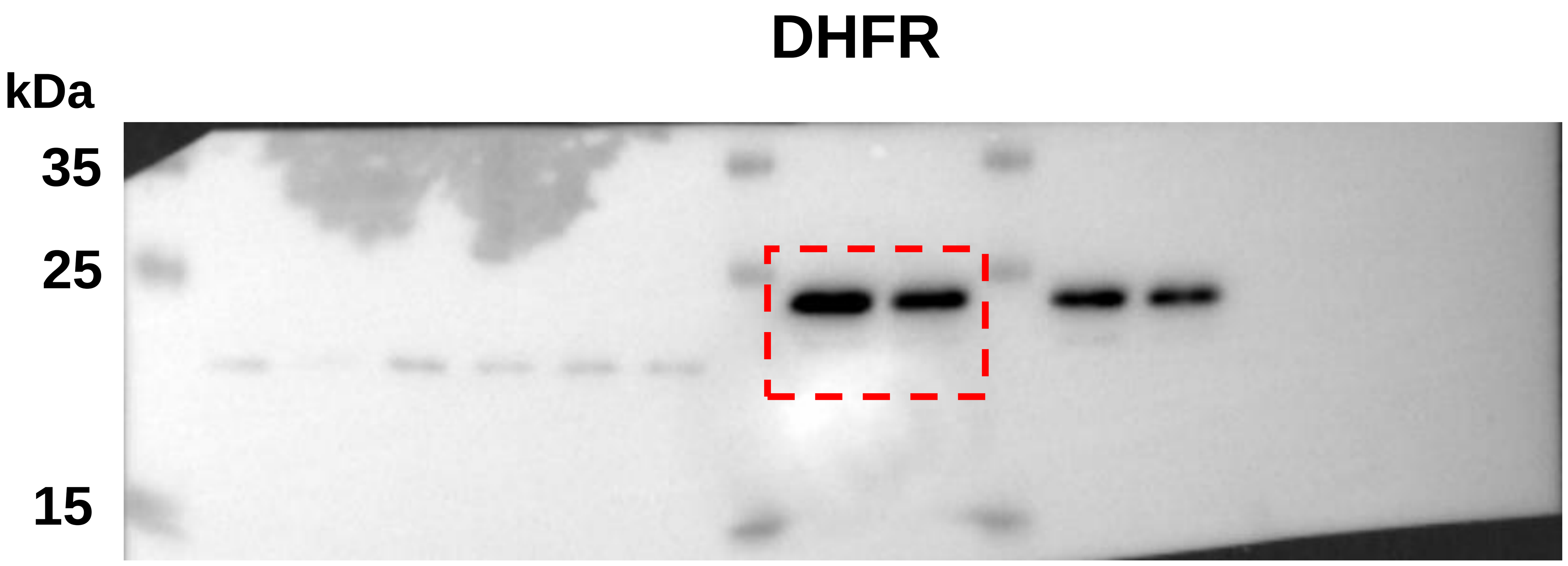

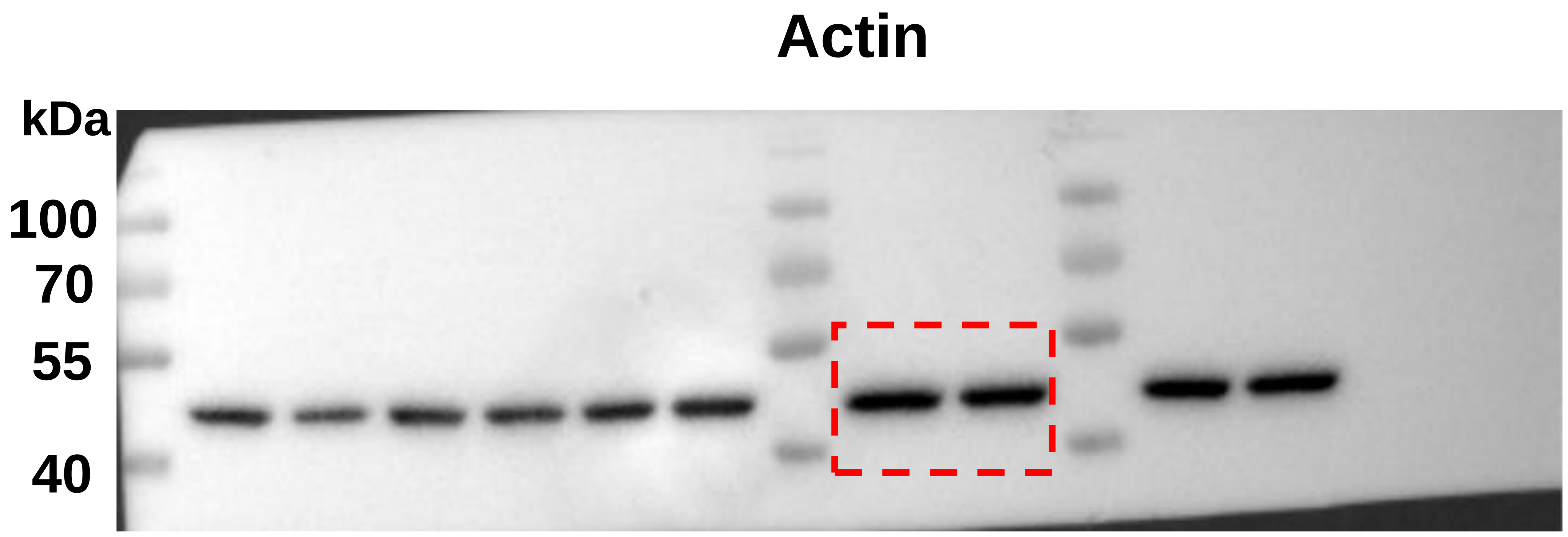

Fig. 3E

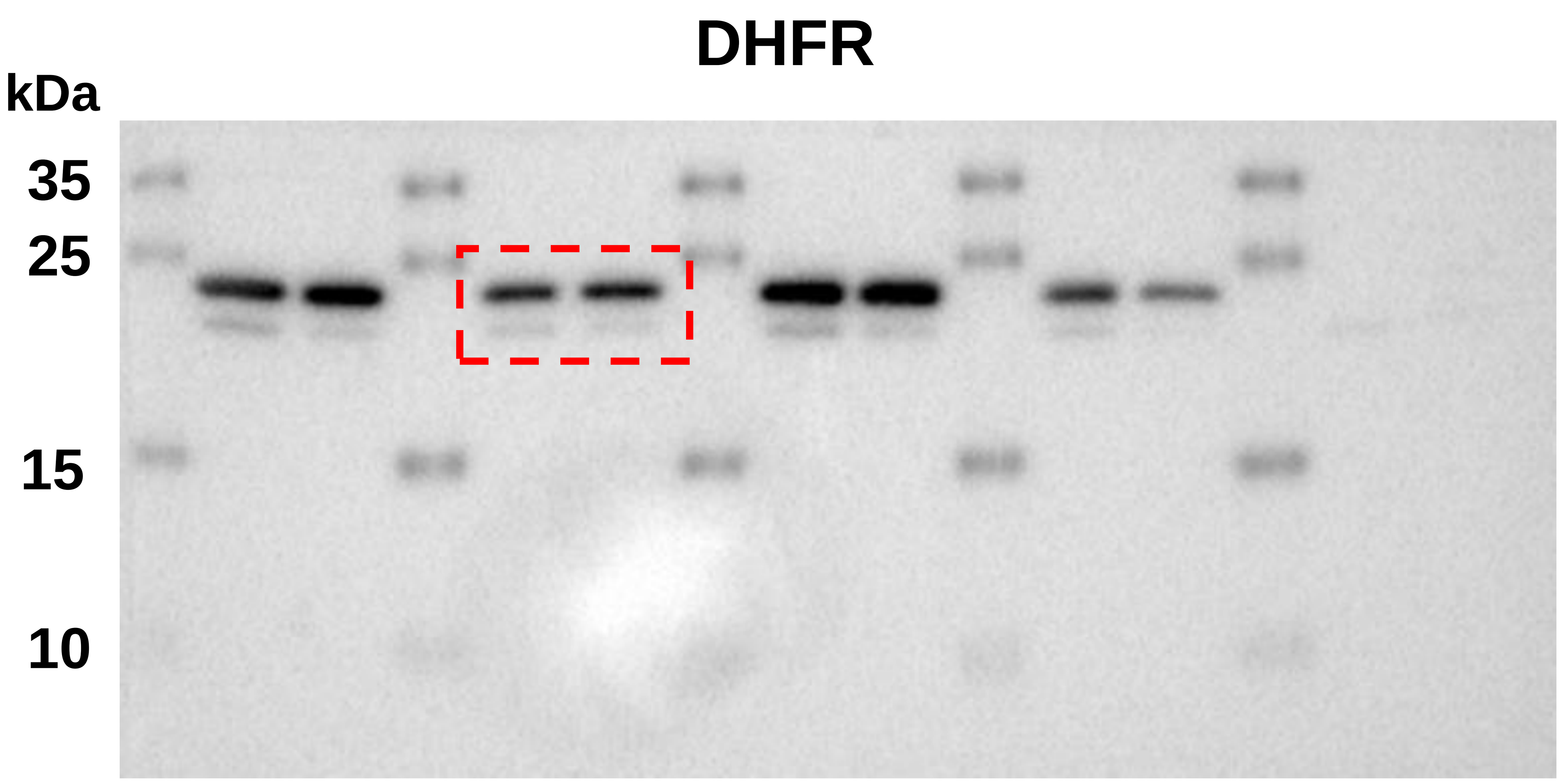

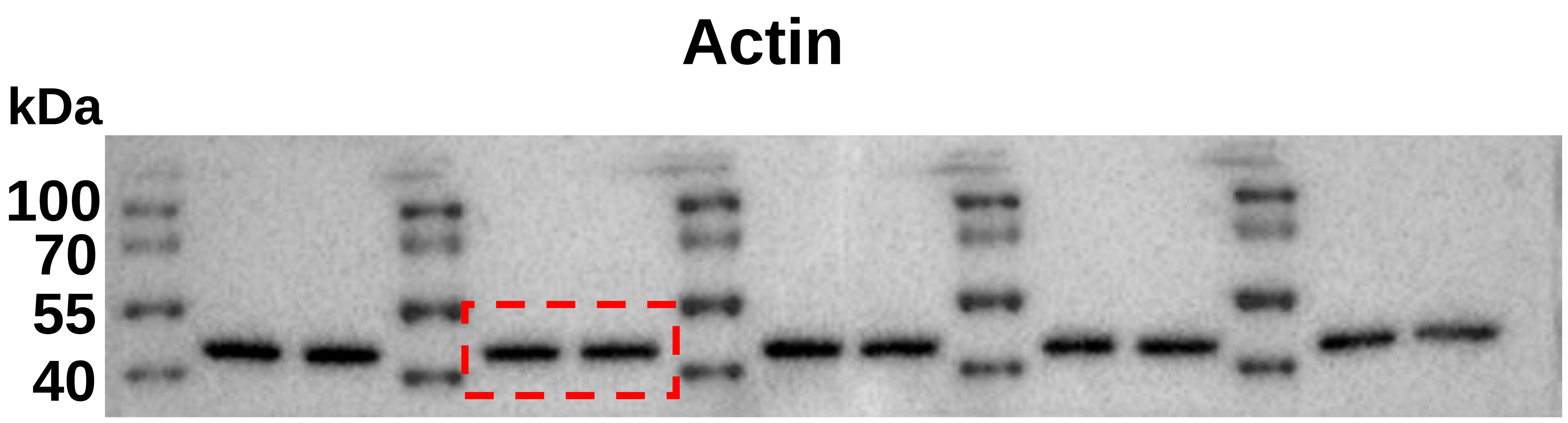

Fig. 5A

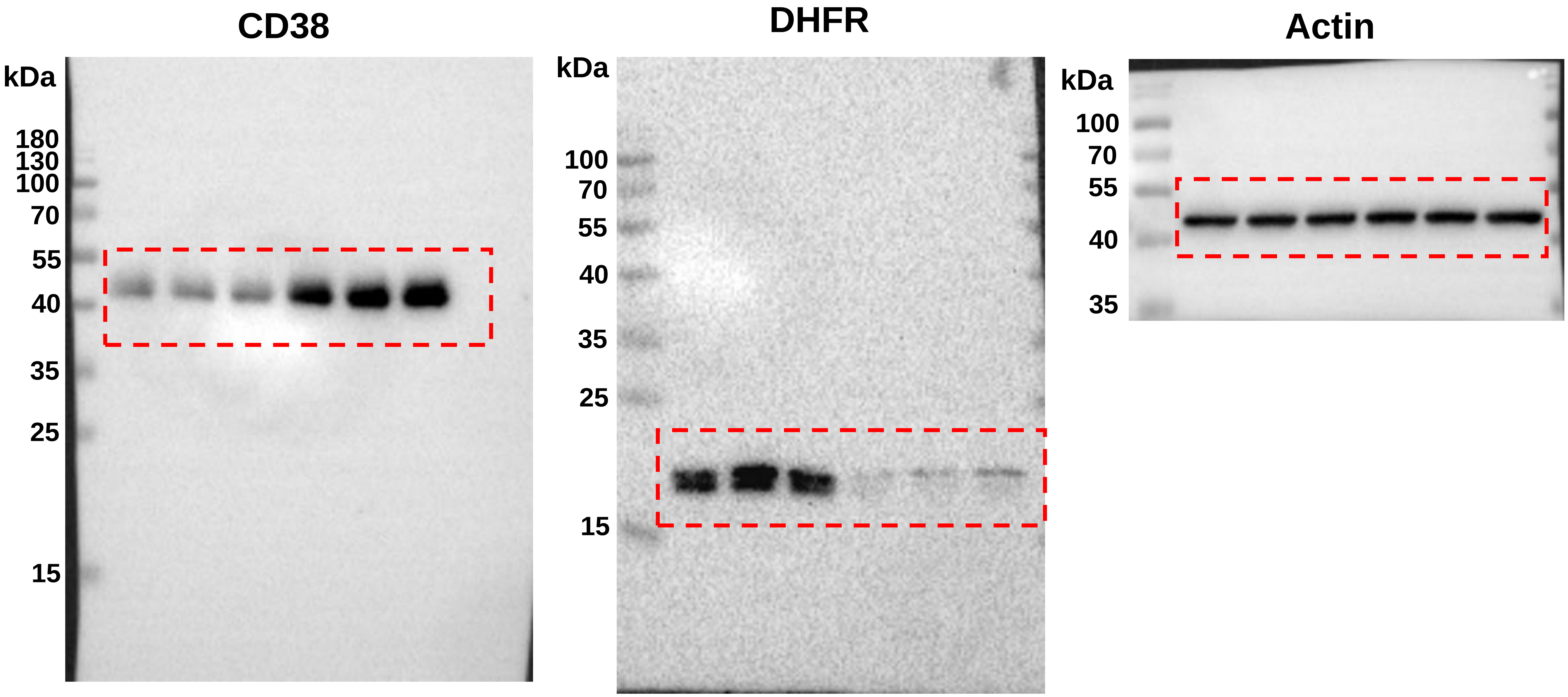
CD38
DHFR
Actin
kDa
180
130
100
70
55
40
35
25
15
kDa
100
70
55
40
35
25
15
kDa
100
70
55
40
35
